# LC-ESI-QTOF/MS Characterization of Phenolic Compounds from Medicinal Plants (Hops and Juniper Berries) and Their Antioxidant Activity

**DOI:** 10.3390/foods9010007

**Published:** 2019-12-20

**Authors:** Jiafei Tang, Frank R. Dunshea, Hafiz A. R. Suleria

**Affiliations:** School of Agriculture and Food, Faculty of Veterinary and Agricultural Sciences, The University of Melbourne, Parkville, VIC 3010, Australia; jiafeit@student.unimelb.edu.au (J.T.); fdunshea@unimelb.edu.au (F.R.D.)

**Keywords:** medicinal plants, hops, juniper berries, polyphenols, liquid chromatography coupled with electrospray-ionization quadrupole time-of-flight mass spectrometry (LC-ESI-QTOF/MS), antioxidant activities

## Abstract

Hops (*Humulus lupulus* L.) and juniper berries (*Juniperus communis* L.) are two important medicinal plants widely used in the food, beverage, and pharmaceutical industries due to their strong antioxidant capacity, which is attributed to the presence of polyphenols. The present study is conducted to comprehensively characterize polyphenols from hops and juniper berries using liquid chromatography coupled with electrospray-ionization quadrupole time-of-flight mass spectrometry (LC-ESI-QTOF/MS) to assess their antioxidant capacity. For polyphenol estimation, total phenolic content, flavonoids and tannins were measured, while for antioxidant capacity, three different antioxidant assays including the 2,2-diphenyl-1-picrylhydrazyl (DPPH) antioxidant assay, the 2,2-azino-bis-3-ethylbenzothiazoline-6-sulfonic acid (ABTS) radical cation decolorization assay and the ferric reducing-antioxidant power (FRAP) assay were used. Hops presented the higher phenolic content (23.11 ± 0.03 mg/g dw) which corresponded to its strong antioxidant activity as compared to the juniper berries. Using LC-ESI-QTOF/MS, a total of 148 phenolic compounds were tentatively identified in juniper and hops, among which phenolic acids (including hydroxybenzoic acids, hydroxycinnamic acids and hydroxyphenylpropanoic acids) and flavonoids (mainly anthocyanins, flavones, flavonols, and isoflavonoids) were the main polyphenols, which may contribute to their antioxidant capacity. Furthermore, the HPLC quantitative analysis showed that both samples had a high concentration of phenolic acids and flavonoids. In the HPLC quantification, the predominant phenolic acids in hops and juniper berries were chlorogenic acid (16.48 ± 0.03 mg/g dw) and protocatechuic acid (11.46 ± 0.03 mg/g dw), respectively. The obtained results highlight the importance of hops and juniper berries as a rich source of functional ingredients in different food, beverage, and pharmaceutical industries.

## 1. Introduction

Medicinal plants are used in different food, beverage, and pharmaceutical industries. They are rich in bioactive compounds especially polyphenols which can contribute to human health. Polyphenols are secondary bioactive compounds which are classified into several categories consisting of hydroxybenzoic acids (protocatechuic, *p*-hydroxybenzoic, syringic), hydroxycinnamic acids (caffeic, *p*-coumaric, ferulic), flavan-3-ols (catechin, epicatechin), flavonoids (catechin, epicatechin, quercetin, apigenin), glycosides, and proanthocyanidins [1]. There is a growing interest in the research of polyphenols due to their antioxidant properties and the evidence for the multiple biological activities, including cardioprotective, anti-inflammatory, anti-carcinogenic, antiviral, and antibacterial properties [2].

Beer is one of the most popular alcoholic beverages in the world [3]. Most of the modern beers are formulated with hops, which contribute bitterness flavors and act as natural preservatives and stabilizers [4]. In addition to hops, some of the brewers normally add a few other flavoring plants such as juniper berries that contain bitter substances, giving the beer a well-rounded, balanced, and tasty bitterness [5]. Hops and juniper berries are rich in phenolic compounds that can contribute to antioxidant capacity and provide a pleasant sensory quality to beverages [4,5,6].

Hops (*Humulus lupulus* L.) have a unique bitterness and aroma, contain various phenolic compounds, and also one of the indispensable raw materials for beer brewing. About 30% of the polyphenols in beer come from these hops. Dried hops cones possess about 15% polyphenols mainly phenolic acids, prenylated chalcone, flavonoids, and catechins [4]. Some of the common polyphenolic substances that are found in different hops varieties are chlorogenic acid, gallic acid, epicatechin, and kaempferol-3-glucoside [7].

Juniper berries (*Juniperus communis* L.) have been used as medicinal plants to treat opportunistic infections, a spice for various cuisines, and distinctive flavoring compounds for different beverages [8]. Previous studies have shown that polyphenols in the juniper berries mainly include flavonoids and bioflavonoids that have antioxidant activities, which could scavenge free radicals, prevent the free radical formation and prevent lipid peroxidation [9]. Some of the phenolic compounds such as epicatechin, procyanidin dimer, and epigallocatechin have been determined by liquid chromatography-mass spectrometer (LC-MS) in the *Juniperus* species found in Portugal [10]. However, relatively less information is available regarding their phenolic profile and antioxidant capacity.

The antioxidant activity of these polyphenols can be assessed by scavenging free radicals or delaying the generation of free radicals using different in vitro methods, including the 2,2-diphenyl-1-picrylhydrazyl (DPPH) antioxidant assay, the 2,2-azino-bis-3-ethylbenzothiazoline-6-sulfonic acid (ABTS) radical cation decolorization assay, and the ferric reducing-antioxidant power (FRAP) assay [11]. Antioxidant capacity can vary depending upon the sample and the nature and type of solvent extraction. Different types of solvents and their combinations have been used for the extraction of polyphenols from plant materials. Water, aqueous mixtures of ethanol, methanol, and acetone are commonly used solvents to extract compounds with high extraction yields [12]. After an extraction, precise identification and quantitation of these phenolic compounds is a complex task because of their structural diversity.

Liquid chromatography coupled with electrospray-ionization quadrupole time-of-flight mass spectrometry (LC-ESI-QTOF/MS) has been recognized as a powerful analytical tool with high sensitivity and accuracy to determine the phenolic profile of plant materials [13]. Also, high-pressure liquid chromatography (HPLC) has proven to be a very useful tool in the quantitation of targeted polyphenols, in combination with different detectors like ultraviolet–visible (UV) and photodiode array detector (PDA) [2]. These analytical techniques are considered as advanced tools for the characterization, purification, and quantitation of phenolic compounds.

Hops and juniper berries are very important medicinal plants that have strong antioxidant capacity. Therefore, the objective of our study was to identify and characterize the polyphenols from selected medical plants (hops and juniper berries) using LC-ESI-QTOF/MS and quantify through HPLC-PDA. Another objective was to measure the total phenolic content (TPC), total flavonoid content (TFC), and total tannin content (TTC) and further analyze their antioxidant capacity using DPPH, FRAP, and ABTS radical-scavenging activity.

## 2. Materials and Methods

### 2.1. Chemical and Reagents

Most of the chemicals used for extraction and characterization were analytical grade and purchased from Sigma-Aldrich (Castle Hill, NSW, Australia). Folin–Ciocalteu reagent, gallic acid, aluminum chloride hexahydrate, quercetin, vanillin, catechin, 2,2-diphenyl-1-picrylhydrazyl (DPPH), ascorbic acid, 2, 4, 6-tripyridyl-s-triazine (TPTZ), HCl, 2, 2’-azino-bis(3-ethylbenzothiazoline-6-sulfonic acid) and potassium persulfate were purchased from Sigma-Aldrich (St. Louis, MO, USA). Sodium carbonate, ethanol, sodium acetate, sulfuric acid, ferric chloride (Fe [III]Cl_3_·6H_2_O), and acetic acid were purchased from the Thermo Fisher (Scoresby, Melbourne, VIC, Australia). HPLC grade methanol, acetic acid, and acetonitrile used for HPLC analyses were purchased from Sigma-Aldrich (St. Louis, MO, USA). Phenolic acid and flavonoid standards (gallic acid, protocatechuic acid, caftaric acid, *p*-hydroxybenzoic acid, chlorogenic acid, caffeic acid, syringic acid, coumaric acid, catechin, epicatechin gallate, quercetin-3-galactoside, quercetin-3-*O*-glucuronide (q-3-*O*-glucuronide), kaempferol-3-*O*-glucoside, quercetin, and kaempferol) were purchased from Sigma-Aldrich (St. Louis, MO, USA).

### 2.2. Sample Preparation

Dried hops pellets (AlphAroma) were purchased from a local retail market in Melbourne, Australia. Dried juniper berries (Easten red cedar) were purchased from the Ozspice Store, Melbourne, Australia. Hops pellets and juniper berries were milled into dried powder and were stored at room temperature in a dark area to protect from light exposure, prior to an extraction.

### 2.3. Extraction of Phenolic Compounds

Two grams of dried powder of hops and juniper berries were macerated in 20 mL of 30% ethanol (*w*/*v*). The extraction was carried out in a shaking incubator (ZWYR-240, Labwit, Ashwood, VIC, Australia) at 120 rpm, 4 °C for 12 h. Samples were centrifuged (ROTINA 380R centrifuge, Hettich, Victoria, Australia) at 5000 rpm for 15 min. The supernatant was collected and stored at −20 °C for further analysis.

### 2.4. Estimation of Polyphenols and Antioxidant Assays

For polyphenol estimation, TPC, TFC and TTC were measured while for antioxidant capacity, three different antioxidant assays, including DPPH, FRAP, and ABTS, were performed using the method of Gu et al. [14]. The data was obtained by the Multiskan^®^ Go microplate photometer (Thermo Fisher Scientific, Waltham, MA, USA).

#### 2.4.1. Determination of Total Phenolic Content (TPC)

The TPC was determined by a spectrophotometric method using Folin–Ciocalteu reagent [15] with some modifications. For determination, 25 µL of the extract was mixed with 25 µL Folin–Ciocalteu reagent solution (1:3 diluted with water) and 200 µL water was added into a 96-well plate (Corning Inc., Midland, NC, USA) followed by incubation at room temperature for 5 min. The reaction mixture was basified by adding 25 µL 10% (*w:w*) sodium carbonate and incubated again for 60 min in dark area. Then, the absorbance was measured at 765 nm by a spectrophotometer plate reader (Thermo Fisher Scientific, Waltham, MA, USA). The TPC in samples was quantified from a calibration curve prepared with gallic acid standard with different concentrations ranging from 0–200 µg/mL and expressed as mg of gallic acid equivalents (GAE) per g dry weight (dw) (mg GAE/g dw) of the sample.

#### 2.4.2. Determination of Total Flavonoid Content (TFC)

The TFC was determined by the aluminum chloride method [16] with some modifications. An 80 µL of the extract was mixed with 80 µL of 2% aluminum chloride (diluted with ethanol) and 120 µL of 50 g/L sodium acetate solution in a 96-well plate and incubated at 25 °C for 2.5 h. Then, the absorbance of the mixture was subsequently measured at 440 nm. The TFC was calculated as mg of quercetin equivalent per g (mg QE/g dw) of weight of samples using the calibration curve of quercetin (0–50 µg/mL).

#### 2.4.3. Determination of Total Tannins Content (TTC)

The TTC was determined by vanillin and *p*-dimethylaminocinnamaldehyde methods [16] with some modifications. Twenty-five µL of the extract was mixed with 150 µL of 4% vanillin solution (diluted with methanol) and 25 µL of 32% sulfuric acid in a 96-well plate and incubated at 25 °C for 15 min and the absorbance was measured at 500 nm. The TTC was expressed as mg of catechin equivalent per g (mg CE/g dw) of samples using a calibration curve prepared with catechin solution ranging from 0–1000 µg/ML.

#### 2.4.4. 2,2-Diphenyl-1-Picrylhydrazyl (DPPH) Antioxidant Assay

The DPPH scavenging activity was determined by the DPPH assay method [17] with some modifications. For the DPPH. assay, 40 µL of the extract was added to the 40 µL of DPPH methanolic solution (0.1 mM) in a 96-well plate. The mixtures were shaken vigorously and incubated at 25 °C for 30 min and the absorbance was measured at 517 nm. The DPPH radical-scavenging activity of extracts was expressed as mg of ascorbic acid equivalents per g (mg AAE/g dw) of samples using standard equation, plotted at different concentrations of standard ranges from 0–50 µg/mL.

#### 2.4.5. Ferric Reducing-Antioxidant Power (FRAP) Assay

The FRAP assay was determined based on the method [17] with some modifications. The FRAP method involves assessing the ability of the test material to reduce iron in Fe^3+^-TPTZ complex (ferric-2,4,6-tripyridyl-s-Triazine) to the Fe^2+^-TPTZ complex by the test substance [11]. The FRAP dye was prepared by mixing of sodium acetate solution (300 mM), TPTZ (2, 4, 6-tripyridyl-s-triazine) solution (10 mM), and Fe[III] solution (20 mM) in 10:1:1 ratio, respectively. A 20 µL of extract or standard was added to 280 µL of prepared FRAP dye solution in a 96-well plate and incubated at 37 °C for 10 min. The absorbance was measured at 593 nm. The FRAP results were converted to mg of ascorbic acid equivalents per g (mg AAE/g dw) of samples using the standard curve, plotted at different concentrations of standard ranges from 0–50 µg/mL.

#### 2.4.6. 2,2′-Azino-bis-3ethylbenzothiazoline-6-sulfonic Acid (ABTS) Radical Scavenging Assay

The ABTS scavenging activity was carried out by the ABTS^+^ radical cation decolorization assay with some modification [17]. Here, 5 mL of 7 mmol/L of ABTS solution was mixed with 88 µL of a 140 mM potassium persulfate solution to produce ABTS^+^. The mixture was placed in the dark at room temperature for 16 h. Then, the prepared ABTS^+^ solution was diluted with analytical grade ethanol to obtain an initial absorbance of 0.7 at 734 nm. Then, 10 µL of extract or standard was mixed with 290 µL of prepared diluted ABTS solution in a 96-well plate and incubated at room temperature for 6 min in the dark area. Then, the absorbance was measured at 734 nm after incubation. The antioxidant ability was expressed as mg of ascorbic acid equivalents per g (mg AAE/g dw) of samples using the calibration curve prepared for ascorbic acid, plotted at different concentrations of standard ranges from 0–2000 µg/mL.

### 2.5. LC-ESI-QTOF/MS Analysis

Polyphenol characterization was carried out using the method of Gu et al. [14] and was performed by Agilent 1200 series HPLC (Agilent Technologies, CA, USA) equipped with an Agilent 6520 Accurate-Mass Q-TOF LC/MS (Agilent Technologies, CA, USA). The separation was carried out using a Synergi Hydro-RP 80A, LC column 250 × 4.6 mm, 4 µm (Phenomenex, Torrance, CA, USA). Mobile phase A was prepared in the ratio of water/acetic acid (98:2, *v/v*), and mobile phase B consisted of acetonitrile/water/acetic acid (100:1:99, *v/v/v*). Both mobile phase A and B were degassed at 21 °C for 15 min. The extract was filtered using the syringe filters (Kinesis, Redland, QLD, Australia) and transferred into HPLC vials. The flow rate was set to be 0.8 mL/min and the injection volume was 6 μL of each sample. Gradient elution was performed by a mixture of mobile phase A and B in the following program: 0–20 min, 10% B; 20–30 min, 25% B; 30–40 min, 35% B; 40–70 min, 40% B; 70–75 min, 55% B; 75–77 min, 80% B; 77–79 min, 100% B; 79–82 min, 100% B; 82–85 min, 10% B. At the end of program, the eluent composition was back to initial gradient and the column was equilibrated for 3 min before next injection.

Electrospray ionization (ESI) was used as a source in operating both negative and positive modes. Mass spectra in the *m/z* range 50 to 1300 were obtained. The mass spectrometry conditions were set as follows: nitrogen gas temperature 300 °C with the flow rate 5 L/min, sheath gas temperature 250 °C with the flow rate 11 L/min, nebulizer gas pressure 45 psi. The capillary and nozzle voltage were set at 3.5 kV and 500 V, respectively. Data acquisition and analysis were performed using Agilent LC-MS-QTOF MassHunter data acquisition software version B.03.01.

### 2.6. HPLC Analysis

The quantification of targeted phenolic compounds was determined by using the method of Gu et al. [14] and carried by Agilent 1200 series HPLC (Agilent Technologies, CA, USA) equipped with a photodiode array (PDA) detector. The same column and conditions were maintained described above in LC-ESI/QTOF/MS except for sample injection volume of 20 μL. Detection was examined by three different wavelengths (280, 320, and 370 nm) for various phenolic compounds. 280 nm wavelength was used for the identification of hydroxybenzoic acids, 320 nm was used for hydroxycinnamic acids, and 370 nm was used for the identification of the flavonol group. Data acquisition and analysis were performed using Agilent LC-ESI/QTOF/MS MassHunter data acquisition software version B.03.01.

### 2.7. Statistical Analysis

One-way analysis of variance (ANOVA) was used to test for differences in mean values between different samples, followed by Tukey’s honestly significant differences (HSD) multiple rank test at *p* < 0.05. The results are shown as mean ± standard deviation (SD). ANOVA was performed by Minitab Program for Windows version 18.0 (Minitab, LLC, State College, PA, USA).

## 3. Results and Discussion

### 3.1. Polyphenol Estimation (TPC, TFC, and TTC)

Medicinal plants including hops and juniper berries are rich in phenolic compounds. The amount of phenolics content in both samples were determined by the TPC, TFC, and TTC, and the results are expressed as gallic acid equivalents, quercetin equivalent, and catechin equivalent, respectively.

Table 1 shows that the TPC values was significantly higher in hops (23.11 ± 0.03 mg GAE/g dw) as compared to juniper berries (9.08 ± 0.01 mg GAE/g dw; *p* < 0.05). In the present study, it was found that hops and juniper berries had lower TPC values compared with the previously reported studies, which could be due to the fact that researchers applied ethanol extraction with high concentrations [18] or employed a freeze-drying step before extraction [10]. Regarding the TFC, the juniper (2.25 ± 0.01 mg QE/g dw) contains more flavonoids as compared to hops (1.37 ± 0.01 mg QE/g dw). Previously, only a few flavonoids were detected in hops [7] while flavonoids were considered as the major polyphenol classes in juniper berries [9]. Nasri et al. [19] also reported a higher concentration of total flavonoids (8.90 ± 0.48 mg/g dw) from juniper berries (*Juniperus phoenicea*), it was found that different juniper varieties have different flavonoids content. Furthermore, Miceli et al. [20] also reported a significant difference (*p* < 0.05) in TPC and TFC values between different varieties of juniper berries including *J. communis* L. var. *communis* and *J. communis* L. var. *saxatilis* (Pall). Table 1 shows that hops contained a significantly higher (*p* < 0.05) amount of tannins as compared to juniper berries. High tannins in our study are in agreement with previous research of Gorjanović et al. [21] in ethanol extracts of hop (*Humulus lupulus* L.).

### 3.2. Antioxidant Activities

Antioxidant properties are very important due to the deleterious role of free radicals in foods and biological systems [22]. The antioxidant capacities of extracts were evaluated by the most commonly used antioxidant assays: DPPH, FRAP, and ABTS methods.

The DPPH is a stable and purple free radical that has been widely employed to determine antioxidant capacity and presents a typical absorption band at 517 nm [23]. The method is based on the reduction of the stable free radical DPPH^.^ in the presence of a hydrogen-donating antioxidant, and the formation of the non-radical form DPPH-H as a result of the reaction [22]. Table 1 shows that the free radical scavenging activities of hops extract (9.26 ± 0.02 mg AAE/g dw) was significantly higher (*p* < 0.05) than that of juniper extract, which was consistent with the result of TPC, indicating that antioxidant activities of samples were related to the TPC. Previously, Elmastaş et al. [24] reported that juniper ethanol extracts (*J. communis* L.), have stronger DPPH scavenging activity than aqueous extracts. Al-Mustafa et al. [25] also presented a high correlation (*R*^2^ = 0.87) between the DPPH scavenging activity and the total phenolic content of different medicinal extracts.

The ferric reducing power determination is based on the reduction of Fe^3+^ to Fe^2+^ by electron transfer from the sample or antioxidant and the ability of the extracts to act as antioxidants by donating electrons could increase with increased absorbance [22]. Table 1 shows that there was a significant difference (*p* < 0.05) in FRAP values between hops and juniper berries, agreeing with the result of TPC, indicating ferric reducing power of samples may be related to the TPC. Previously, Abram et al. [26] reported simlar FRAP antioxidant capacity in different hops varieties using ethanol extraction. In addition, ethanol extract of *J. communis* showed better scavenging activity in the FRAP assay among five different *Juniperus* species, including *J. communis*, *J. excelsa*, *J. foetidissima*, *J. oxycedrus*, and *J. sabina* [27].

To evaluate the antioxidant capacity of food extracts, ABTS^+^ radical scavenging activity has been widely applied based on hydrogen-donating antioxidants against nitrogen radicals [28]. Table 1 shows that the hops (49.54 ± 0.04 mg AAE/g dw) had significantly higher ABTS^+^ radical scavenging activity as compared to the juniper berries (15.18 ± 0.02 mg AAE/g dw). The ABTS values of our juniper berries differ from the studies of Höferl et al. [8], who reported higher ABTS radical scavenging activity for another juniper berry variety using a different solvent extract. However, Kowalczyk et al. [18] reported similar ABTS^+^ scavenging activity in hops samples extracted with different solvents including aqueous methanol, aqueous ethanol, and water extract.

### 3.3. Phenolic Identification by LC-ESI-QTOF/MS

The LC-ESI-QTOF/MS has been proved to be an effective tool for tentatively identifying and characterizing phenolic compounds in several plants [13]. Identification and characterization of compounds were carried out by comparison of their retention time (RT), mass error between mass observed, and the theoretical mass (<10 ppm); mass spectrometric (MS) data obtained under both negative and positive electron spray ionization modes (ESI^−^/ESI^+^; Supplementary Figures S1 and S2) and data identification scores selected were above 80. Table 2 reports all compounds tentatively identified in both hops and juniper berries in positive and negative ionization modes.

A total of 148 different phenolic compounds were characterized in both hops and juniper berries, including 34 phenolic acids, 78 flavonoids, 8 lignans, 3 stilbenes, 1 hydroxybenzaldehyde, and 24 other polyphenols. Additionally, one non-phenolic metabolite (1,3,5-trimethoxybenzene) was also characterized in hops and juniper berries.

In hops, a total of 117 phenolic compounds were identified (Supplementary Table S1) and categorized into six polyphenol classes including 30 phenolic acids, 61 flavonoids, 4 lignans, 2 stilbenes, 1 hydroxybenzaldehydes, and 19 other polyphenols. In juniper berries, a total of 81 compounds in 5 different classes were characterized (Supplementary Table S2), including 18 phenolic acids, 46 flavonoids, 5 lignans, 2 stilbenes, and 10 other polyphenols.

#### 3.3.1. Hydroxybenzoic Acids

In our study, a total of 3 hydroxybenzoic acids derivatives (Compounds **3**, **4** and **6**) were identified in both hops and juniper berries in negative modes of ionization. Compound **3** with [M − H]^−^ at *m/z* 315.0746 and 315.0739 was tentatively characterized as protocatechuic acid 4-*O*-glucoside. Compound **4** with [M − H]^−^ at *m/z* 153.0203 and 153.0204 was tentatively identified as 2,3-dihydroxybenzoic acid. Compound **6** with the molecular formula C_7_H_6_O_3_ and having the precursor ion at *m/z* 137.0249 and 137.0258 in the negative ESI-mode, was tentatively characterized as 2-hydroxybenzoic acid in both hops and juniper berries. Previously, protocatechuic acid 4-*O*-glucoside and 2-hydroxybenzoic had already been identified in *Juniperus communis* var. saxatilis [29].

Compounds **1** and **2**, only detected in hops, with [M − H]^−^ at *m/z* 331.0693 and 169.0159 were tentatively characterized as galloyl glucose and gallic acid, respectively, while there were two compounds (Compounds **5** and **7**) detected only in juniper berries in negative ionization modes and tentatively assigned as 4-*O*-methylgallic acid and ellagic acid with [M − H]^−^ at *m/z* 183.0306 and 300.9969, respectively. Previously Miceli et al. [30] also found gallic acid present in *J. drupacea* berries methanol extract by HPLC-DAD-MS. Also, the gallic acid was previously reported in *Humulus lupulus* L. by HPLC-UV [31].

#### 3.3.2. Hydroxycinnamic Acids

In the present work, we characterized 6 hydroxycinnamic acids (Compounds **9**, **11**, **16**, **19**, **22**, and **23**) in both hops and juniper berries. Among these, two compounds (Compounds **9** and **19**) were identified in both hops and juniper berries samples in positive and negative modes of ionization. Compound **9** with [M + H]^+^ at *m/z* 399.1291 and [M − H]^−^ at *m/z* 397.1117 was tentatively characterized as 3-sinapoylquinic acid while compound (**19**) with [M − H]^−^ at *m/z* 326.1042 and [M + H]^+^ at *m/z* 328.1172 was tentatively identified as *p*-coumaroyl tyrosine. Two more compounds (Compounds **11** and **22**) were detected in both samples in negative ionization modes. Compound **11** with [M − H]^−^ at *m/z* 337.0949 and 337.0955 was tentatively identified as 3-*p*-coumaroylquinic acid while compound **22** showing [M − H]^−^ at *m/z* 191.0733 and 191.072 was tentatively identified as *p*-coumaric acid ethyl ester. However, two compounds (Compounds **16** and **23**) were identified in both samples in positive modes of ionization. Compound **16** with [M + H]^+^ at *m/z* 149.0587 and 149.0591 (at RT = 20.491 min) was tentatively characterized as cinnamic acid while compound **23** with [M + H]^+^ at *m/z* 195.0656 and 195.0668 was tentatively identified as isoferulic acid. In addition, isoferulic acid and *p*-coumaric acid ethyl ester were previously reported in *Juniperus communis* var. saxatilis [29].

In addition to the compounds identified above in both plant samples, there were a total of 9 hydroxycinnamic acids derivatives characterized only in hops, including 3-caffeoylquinic acid, caffeic acid 3-*O*-glucuronide, rosmarinic acid, *p*-coumaric acid 4-*O*-glucoside, ferulic acid 4-*O*-glucuronide, 3-feruloylquinic acid, ferulic acid 4-*O*-glucoside, caffeoyl glucose, and 1,2-disinapoylgentiobiose. The *p*-coumaric acid ethyl ester and 3-caffeoylquinic acid were reported as the bioactive compounds in Saaz hops variety from the Czech Republic [32].

#### 3.3.3. Hydroxyphenylpropanoic Acids

There were 2 hydroxyphenylpropanoic acids (Compounds **33** and **34**) detected in both hops and juniper berries. Compound **33** with [M − H]^−^ at *m/z* 181.0524 and with [M − H]^−^ at *m/z* 181.0524 was tentatively identified as 3-hydroxy-3-(3-hydroxyphenyl) propionic acid. Also, compound **34** showing [M − H]^−^ at *m/z* 165.0569 and 165.0555 and with the molecular formula C_9_H_10_O_3_ was tentatively characterized as 3-hydroxyphenylpropionic acid. Based on QTOF-MS analysis, compounds **30**, **31** and **32** were only detected in hops and tentatively identified as dihydrocaffeic acid 3-*O*-glucuronide, dihydrosinapic acid, and dihydroferulic acid 4-*O*-glucuronide, showing [M − H]^−^ at *m/z* 357.0831, 225.076, and 371.0962, respectively.

#### 3.3.4. Anthocyanins

Based on MS data, a total of 4 anthocyanins were identified in both hops and juniper berries in negative ionization modes, including 2 cyanidin derivatives (Compounds **41** and **48**) and 2 delphinidin 3-*O* derivatives (Compounds **36** and **44**). Compound **48** with [M − H]^−^ at *m/z* 448.0982 and 448.0985 was tentatively characterized as cyanidin 3-*O*-galactoside while compound **41** showing [M − H]^−^ at *m/z* 610.1530 and 610.1529 was tentatively identified as cyanidin 3,5-*O*-diglucoside, all of which were cyanidin derivatives. Additionally, compound **44** showing 39.382 min was tentatively characterized as delphinidin 3-*O*-glucoside, while compound **36** with the molecular formula C_27_H_31_O_17_ was tentatively identified as delphinidin 3-*O*-glucosyl-glucoside, which belonged to delphinidin 3-*O* derivatives.

There were 12 compounds (**35**, **37**, **38**, **39**, **40**, **42**, **43**, **45**, **46**, **47**, **50**, and **51**) only identified in hops in negative ionization modes, mostly being cyanidin and its 3-*O*-glycosides. In juniper berries, compound **49** was tentatively characterized for cyanidin 3-*O*-(6’’-dioxalyl-glucoside) with [M − H]^−^ at *m/z* 591.0656 and 45.432 min.

#### 3.3.5. Flavones

In the present work, we identified 2 flavones (Compounds **71** and **73**) in both hops and juniper berries in negative modes of ionization. Compound **71** was detected in negative ionization modes with [M − H]^−^ at *m/z* 593.1532 and 593.1518 and was tentatively characterized as apigenin 6,8-di-C-glucoside. In addition, compound **73** showed [M − H]^−^ at *m/z* 447.0949 and 447.0941 and was tentatively identified as 6-hydroxyluteolin 7-*O*-rhamnoside, which was also discussed in previously literature in *Juniperus communis* var. saxatilis [29]. Apigenin was previously identified in Tuscan berries of *Juniperus communis* L. by HPLC/DAD/ESI/MS [9].

Compound **78** was only detected in hops with a precursor ion at [M + H]^+^ *m/z* 359.1116, tentatively representing the gardenin B. In juniper berries, compounds **70**, **76,** and **77** with [M + H]^+^ at *m/z* 579.1675, 565.1538, and 345.0957, respectively, were tentatively characterized as isorhoifolin, apigenin 7-*O*-apiosyl-glucoside, and cirsilineol.

#### 3.3.6. Flavonols

In this work, a total of 13 flavonols were detected in both hops and juniper berries in positive and negative ionization modes, including 2 isorhamnetin derivatives (Compounds **94** and **99**), 5 kaempferol derivatives (Compounds **79, 81, 83, 88**, and **89**), 4 myricetin derivatives (Compounds **82**, **84**, **85**, and **93**), and 2 quercetin 3-*O* derivatives (Compounds **90** and **96**). Compound **99** with [M − H]^−^ at *m/z* 315.0508 and 315.0520 was tentatively characterized as isorhamnetin. In previously literature, isorhamnetin was already reported in the Saaz hops variety [33]. Among kaempferol derivatives, compound **83** showing [M + H]^+^ at *m/z* 757.2133 was observed and tentatively identified as kaempferol 3-*O*-glucosyl-rhamnosyl-galactoside. Regarding myricetin derivatives, compound **85** showed [M + H]^+^ at *m/z* 319.0427 and 319.0435, and at 33.345 min and was tentatively characterized as myricetin.

In hops compounds **86**, **95**, **97**, and **98** with [M − H]^−^ at *m/z* 741.1900, 549.0901, 635.1637, and 533.0944, were tentatively identified to be quercetin 3-*O*-xylosyl-rutinoside, quercetin 3-*O*-(6”-malonyl-glucoside), kaempferol 3-*O*-(6’’-acetyl-galactoside) 7-*O*-rhamnoside, and 5,4’-dihydroxy-3,3’-dimethoxy-6:7-methylenedioxyflavone 4’-*O*-glucuronide, respectively. In juniper berries compounds **80** and **92** were detected in negative modes of ionization and tentatively characterized as patuletin 3-*O*-glucosyl-(1->6)-[apiosyl(1->2)]-glucoside and spinacetin 3-*O*-glucosyl-(1->6)-glucoside with precursor [M − H]^−^ at *m/z* 787.1965 and 669.1689, respectively.

#### 3.3.7. Isoflavonoids

A total of 4 isoflavonoids (Compounds **104**, **105**, **107,** and **111**) were detected in both hops and juniper berries. Among which, in positive and negative ionization modes, compound **111** with [M + H]^+^ at *m/z* 301.1069 and with [M − H]^−^ at *m/z* 299.0931, respectively, was tentatively identified as sativanone. In addition, in negative ionization modes, compound **107** with [M − H]^−^ at *m/z* 301.0375 and 301.0364 and at 69.083 min was tentatively characterized as 5,6,7,3’,4’-pentahydroxyisoflavone, which was also detected in the Saaz hops variety [32] and *Juniperus communis* var. saxatilis [29] in previous literature.

Compounds **101**, **102,** and **108** were detected only in hops giving [M + H]^+^ at *m/z* 303.0847, 459.1279 and 285.0766 and tentatively characterized as 4’-methoxy-2’,3,7-trihydroxyisoflavanone, 6’’-*O*-acetyldaidzin and 2’-hydroxyformononetin, respectively. In juniper berries compounds **100**, **103**, **109**, **110**, and **112** were tentatively identified as 6’’-*O*-acetylgenistin, 3’-hydroxydaidzein, 2’,7-dihydroxy-4’,5’-dimethoxyisoflavone, 3’-hydroxymelanettin, and dihydrobiochanin A in positive ionization modes, respectively.

### 3.4. HPLC Analysis

The HPLC is used to study the polyphenol content and chemical composition of various plants, which had been previously shown to be an effective technique for the quantification of polyphenols [2]. In our study, 15 targeted polyphenols mainly phenolic acids and flavonoids were quantified by their UV spectra and by comparing their retention times with reference standards. According to the HPLC-PDA, flavonoids were the main phenolic class with a higher diversity of compounds (Table 3).

Phenolic acids were high in both hops and juniper berries, representing the dominant class of compounds in these selected medicinal plants. In general, chlorogenic acid (16.48 ± 0.03 mg/g dw), gallic acid (3.41 ± 0.02 mg/g dw), caftaric acid (0.72 ± 0.01 mg/g dw), and syringic acid (0.03 ± 0.01 mg/g dw) were the major phenolic acids in hops. However, these compounds were not detected in juniper berries which contained high concentrations of protocatechuic acid (11.46 ± 0.03 mg/g dw), *p*-hydroxybenzoic acid (3.12 ± 0.01 mg/g dw), and caffeic acid (0.14 ± 0.01 mg/g dw) in comparison with those in hops. Coumaric acid was present only in juniper berries with (0.32 ± 0.01 mg/g dw). Previously, chlorogenic acid and gallic acid were determined in hops (*Humulus lupulus* L.) with a high content using HPLC [7]. Previously, Keskin et al. [31] quantified the coumaric acid in *Humulus lupulus* L. using multiple extractions of diethyl ether, ethyl acetate, and methanol. Chlorogenic acid was also reported in *Juniperus communis* L. one of the native species grown on Romanian southern sub-Carpathian hills using 50% ethanol (*w/v*) [34]. Also, protocatechuic acid and gallic acid were quantitated by HPLC in *Juniperus drupacea* berries from Turkey [30].

Regarding flavonoids, catechin was the most abundant flavonoid in both hops and juniper berries with (9.03 ± 0.02 dw) and (8.47 ± 0.02 dw), respectively. In a small amount, epicatechin gallate was determined in hops (0.02 ± 0.01 mg/g dw) but absent in juniper berries. Quercetin-3-*O*-galactoside and kaempferol were higher in juniper berries as compared to hops. Kaempferol-3-*O*-glucoside, quercetin, and quercetin-3-*O*-glucuronide were higher in hops but also detected in juniper berries. In previous literature, catechin, quercetin and kaempferol-3-*O*-glucoside were higher in hops (*Humulus lupulus* L.) while catechin was higher in *J. drupacea* berries from Turkey [30].

## 4. Conclusions

The LC-ESI-QTOF/MS analysis was applied for the tentative identification and characterization of phenolic compounds from hops and juniper berries. Consequently, a total 148 phenolic compounds were tentatively identified, based on comparison of their mass spectrometric data obtained under both negative and positive electron spray ionization conditions and categorized into several main polyphenol classes including phenolic acids, flavonoids, lignans, stilbenes, and other polyphenols. The quantification of 15 individual polyphenols was achieved through HPLC-PDA by comparing their UV spectra and the retention times with reference standards. Different antioxidant assays were conducted to evaluate and map an overall antioxidant capacity of both samples. The results show that hops contain a significantly higher phenolic content and antioxidant capacity compared to juniper berries. In addition, antioxidant capacity is related to phenolic content, which can also be consistent with the presented HPLC composition. Although these two plant species show significant differences in phenolic content, they all present antioxidant capacity, which supports their wide application in health, nutrition, and medicine.

## Figures and Tables

**Table 1:** Polyphenol content and antioxidant activities in hops and juniper berries.

Antioxidant Assays	Hops	Juniper Berries
TPC/mg GAE/g	23.11 ± 0.03 ^a^	9.08 ± 0.01 ^b^
TFC/mg QE/g	1.37 ± 0.01 ^a^	2.25 ± 0.01 ^a^
TTC/mg CE/g	25.18 ± 0.07 ^a^	3.48 ± 0.03 ^b^
FRAP/mg AAE/g	4.17 ± 0.03 ^a^	2.02 ± 0.01 ^b^
DPPH/mg AAE/g	9.26 ± 0.02 ^a^	3.57 ± 0.01 ^b^
ABTS/mg AAE/g	49.54 ± 0.04 ^a^	15.18 ± 0.02 ^b^

All data are the mean ± SD of three replicates. Means followed by different letters (a, b) within the same column are significantly different (*p* < 0.05) from each other. Data of hops and juniper berries are reported on a dry weight basis.

**Table 2:** Qualitative characterization of phenolic compounds in hops and juniper berries by LC-ESI-QTOF/MS.

No.	Proposed Compounds	Molecular Formula	Retention Time (min)	Mode of Lonization (ESI−/ESI+)	Molecular Weight	Theoretical (*m/z*)	Observed (*m/z*)	Mass Error (ppm)	Samples
**Phenolic acids**									
	**Hydroxybenzoic acids**								
1	Galloyl glucose	C_13_H_16_O_10_	6.583	[M − H]^−^	332.0743	331.0670	331.0693	6.70	Hops
2	Gallic acid	C_7_H_6_O_5_	6.749	[M − H]^−^	170.0215	169.0142	169.0159	9.68	Hops
3	Protocatechuic acid 4-*O*-glucoside	C_13_H_16_O_9_	9.151	[M − H]^−^	316.0794	315.0721	315.0746	7.97	* Hops, juniper berries
4	2,3-Dihydroxybenzoic acid	C_7_H_6_O_4_	12.348	[M − H]^−^	154.0266	153.0193	153.0203	6.14	* Hops, juniper berries
5	4-*O*-Methylgallic acid	C_8_H_8_O_5_	14.439	[M − H]^−^	184.0372	183.0299	183.0306	4.71	Juniper berries
6	2-Hydroxybenzoic acid	C_7_H_6_O_3_	19.935	[M − H]^−^	138.0317	137.0244	137.0249	3.69	* Hops, juniper berries
7	Ellagic acid	C_14_H_6_O_8_	45.283	[M − H]^−^	302.0063	300.9990	300.9969	−7.08	Juniper berries
	**Hydroxycinnamic acids**								
8	3-Caffeoylquinic acid	C_16_H_18_O_9_	12.629	[M − H]^−^	354.0951	353.0878	353.0894	1.84	Hops
9	3-Sinapoylquinic acid	C_18_H_22_O_10_	13.815	[M − H]^−^/* [M + H]^+^	398.1213	399.1286	399.1291	0.88	* Hops, juniper berries
10	Caffeic acid 3-*O*-glucuronide	C_15_H_16_O_10_	15.396	[M − H]^−^	356.0743	355.0670	355.0680	3.79	Hops
11	3-*p*-Coumaroylquinic acid	C_16_H_18_O_8_	17.665	[M − H]^−^	338.1002	337.0929	337.0949	6.22	* Hops, juniper berries
12	Rosmarinic acid	C_18_H_16_O_8_	17.665	[M − H]^−^	360.0845	359.0772	359.0780	8.44	Hops
13	*p*-Coumaric acid 4-*O*-glucoside	C_15_H_18_O_8_	18.957	[M − H]^−^	326.1002	325.0929	325.0920	−4.31	Hops
14	Ferulic acid 4-*O*-glucuronide	C_16_H_18_O_10_	19.918	[M − H]^−^	370.0900	369.0827	369.0833	1.54	Hops
15	3-Feruloylquinic acid	C_17_H_20_O_9_	20.481	[M − H]^−^	368.1107	367.1034	367.1038	0.94	Hops
16	Cinnamic acid	C_9_H_8_O_2_	20.491	[M + H]+	148.0524	149.0597	149.0587	−6.81	* Hops, juniper berries
17	Ferulic acid 4-*O*-glucoside	C_16_H_20_O_9_	22.916	[M − H]^−^	356.1107	355.1034	355.1058	6.08	Hops
18	Caffeoyl glucose	C_15_H_18_O_9_	24.076	[M − H]^−^	342.0951	341.0878	341.0898	6.09	Hops
19	*p*-Coumaroyl tyrosine	C_18_H_17_NO_5_	27.637	* [M − H]^−^/[M + H]+	327.1107	326.1034	326.1042	−3.98	* Hops, juniper berries
20	1,2-Disinapoylgentiobiose	C_34_H_42_O_19_	37.991	[M − H]^−^	754.2320	753.2247	753.2281	−5.47	Hops
21	Verbascoside	C_29_H_36_O_15_	54.046	[M − H]^−^	624.2054	623.1981	623.1982	−0.85	Juniper berries
22	*p*-Coumaric acid ethyl ester	C_11_H_12_O_3_	81.109	[M − H]^−^	192.0786	191.0713	191.0733	9.96	* Hops, juniper berries
23	Isoferulic acid	C_10_H_10_O_4_	81.881	[M + H]+	194.0579	195.0652	195.0656	2.46	*Hops, juniper berries
	**Hydroxyphenylpentanoic acids**								
24	5-(3’-Methoxy-4’-hydroxyphenyl)-gamma -valerolactone	C_12_H_14_O_4_	19.077	[M − H]^−^	222.0892	221.0819	221.0835	7.82	Juniper berries
	**Hydroxyphenylacetic acids**								
25	2-Hydroxy-2-phenylacetic acid	C_8_H_8_O_3_	15.247	[M − H]^−^	152.0473	151.0400	151.0408	6.70	* Hops, juniper berries
26	3,4-Dihydroxyphenylacetic acid	C_8_H_8_O_4_	40.227	[M − H]^−^	168.0423	167.0350	167.0367	9.63	* Hops, juniper berries
	**Hydroxyphenylpentanoic acids**								
27	3-Hydroxyphenylvaleric acid	C_11_H_14_O_3_	8.978	[M + H]^+^	194.0943	195.1016	195.1019	−0.78	Hops
28	5-(3’,4’-dihydroxyphenyl)-valeric acid	C_11_H_14_O_4_	48.883	[M + H]^+^	210.0892	211.0965	211.0956	−4.05	Hops
29	5-(3’,4’,-dihydroxyphenyl)-γ-valerolactone	C_11_H_12_O_4_	68.437	[M − H]^−^	208.0736	207.0663	207.0679	8.32	* Hops, juniper berries
	**Hydroxyphenylpropanoic acids**								
30	Dihydrocaffeic acid 3-*O*-glucuronide	C_15_H_18_O_10_	13.772	[M − H]^−^	358.0900	357.0827	357.0831	1.32	Hops
31	Dihydrosinapic acid	C_11_H_14_O_5_	15.909	[M − H]^−^	226.0841	225.0768	225.0760	−5.07	Hops
32	Dihydroferulic acid 4-*O*-glucuronide	C_16_H_20_O_10_	18.957	[M − H]^−^	372.1056	371.0983	371.0962	−4.96	Hops
33	3-Hydroxy-3-(3-hydroxyphenyl) propionic acid	C_9_H_10_O_4_	48.095	[M − H]^−^	182.0579	181.0506	181.0524	9.61	* Hops, juniper berries
34	3-Hydroxyphenylpropionic acid	C_9_H_10_O_3_	49.139	[M − H]^−^	166.0630	165.0557	165.0569	6.63	* Hops, juniper berries
**Flavonoids**									
	**Anthocyanins**								
35	Cyanidin 3-*O*-(6’’-*p*-coumaroyl-glucoside)	C_30_H_27_O_13_	8.140	[M − H]^−^	595.1452	594.1379	594.1361	−2.65	Hops
36	Delphinidin 3-*O*-glucosyl-glucoside	C_27_H_31_O_17_	32.143	[M − H]^−^	627.1561	626.1488	626.1464	−4.36	* Hops, juniper berries
37	Peonidin 3-*O*-sambubioside-5-*O*-glucoside	C_33_H_41_O_20_	32.640	[M − H]^−^	757.2191	756.2118	756.2098	−2.92	Hops
38	Cyanidin 3-*O*-sambubioside 5-*O*-glucoside	C_32_H_39_O_20_	33.104	[M − H]^−^	743.2035	742.1962	742.1933	−2.55	Hops
39	Pelargonidin 3-*O*-glucosyl-rutinoside	C_33_H_41_O_19_	34.644	[M − H]^−^	741.2242	740.2169	740.2153	−2.68	Hops
40	Delphinidin 3-*O*-sambubioside	C_26_H_29_O_16_	35.903	[M − H]^−^	597.1456	596.1383	596.1363	−2.73	Hops
41	Cyanidin 3,5-*O*-diglucoside	C_27_H_31_O_16_	37.079	[M − H]^−^	611.1612	610.1539	610.1530	−2.01	* Hops, juniper berries
42	Cyanidin 3-*O*-(6’’-malonyl-3’’-glucosyl-glucoside)	C_30_H_33_O_19_	38.189	[M − H]^−^	697.1616	696.1543	696.1524	−2.24	Hops
43	Cyanidin 3-*O*-rutinoside	C_27_H_31_O_15_	38.355	[M − H]^−^	595.1663	594.1590	594.1570	−3.45	Hops
44	Delphinidin 3-*O*-glucoside	C_21_H_21_O_12_	39.382	[M − H]^−^	465.1033	464.0960	464.0945	−3.80	* Hops, juniper berries
45	Peonidin 3-*O*-sophoroside	C_28_H_33_O_16_	41.005	[M − H]^−^	625.1769	624.1696	624.1682	−1.55	Hops
46	Delphinidin 3-*O*-(6’’-acetyl-glucoside)	C_23_H_23_O_13_	42.678	[M − H]^−^	507.1139	506.1066	506.1040	−5.07	Hops
47	Pelargonidin 3,5-*O*-diglucoside	C_27_H_31_ClO_15_	42.844	[M − H]^−^	630.1351	629.1278	629.1293	−0.28	Hops
48	Cyanidin 3-*O*-galactoside	C_21_H_21_O_11_	43.275	[M − H]^−^	449.1084	448.1011	448.0982	−6.42	* Hops, juniper berries
49	Cyanidin 3-*O*-(6’’-dioxalyl-glucoside)	C_25_H_20_O_17_	45.432	[M − H]^−^	592.0700	591.0627	591.0656	4.65	Juniper berries
50	Cyanidin 3-*O*-(6’’-acetyl-glucoside)	C_23_H_23_O_12_	51.143	[M − H]^−^	491.1190	490.1117	490.1083	−5.46	Hops
51	Cyanidin	C_15_H_11_O_6_	79.801	[M − H]^−^	287.0556	286.0483	286.0468	−3.14	Hops
	**Chalcones**								
52	Xanthohumol	C_21_H_22_O_5_	82.941	[M + H]^+^	354.1467	355.1540	355.1523	−3.79	Hops
	**Dihydrochalcones**								
53	3-Hydroxyphloretin 2’-*O*-glucoside	C_21_H_24_O_11_	18.924	[M − H]^−^	452.1319	451.1246	451.1252	−1.17	*Hops, juniper berries
54	Phloridzin	C_21_H_24_O_10_	50.617	[M − H]^−^	436.1369	435.1296	435.1301	1.77	Juniper berries
	**Dihydroflavonols**								
55	Dihydroquercetin 3-*O*-rhamnoside	C_21_H_22_O_11_	26.544	[M − H]^−^	450.1162	449.1089	449.1103	3.17	Hops
56	Dihydromyricetin 3-*O*-rhamnoside	C_21_H_22_O_12_	64.802	[M + H]^+^	466.1111	467.1184	467.1164	−3.04	Hops
	**Flavanols**								
57	Procyanidin dimer B1	C_30_H_26_O_12_	14.932	[M − H]^−^	578.1424	577.1351	577.1355	0.25	* Hops, juniper berries
58	(-)-Epigallocatechin	C_15_H_14_O_7_	16.605	[M − H]^−^	306.0740	305.0667	305.0668	0.88	Hops
59	Procyanidin trimer C1	C_45_H_38_O_18_	18.576	[M − H]^−^	866.2058	865.1985	865.1966	−2.37	* Hops, juniper berries
60	4’-*O*-Methylepigallocatechin	C_16_H_16_O_7_	24.450	[M + H]^+^	320.0896	321.0969	321.0959	−3.17	Hops
61	(-)-Epicatechin	C_15_H_14_O_6_	25.848	* [M − H]^−^/[M + H]^+^	290.0790	289.0717	289.0736	6.06	* Hops, juniper berries
62	4’’-*O*-Methylepigallocatechin 3-*O*-gallate	C_23_H_20_O_11_	26.636	[M + H]^+^	472.1006	473.1079	473.1062	−3.01	Hops
63	Cinnamtannin A2	C_60_H_50_O_24_	29.592	[M − H]^−^	1154.2690	1153.2620	1153.2610	−0.97	Hops
64	(+)-Gallocatechin 3-*O*-gallate	C_22_H_18_O_11_	49.606	[M − H]^−^	458.0849	457.0776	457.0769	0.42	Juniper berries
65	3’-*O*-Methyl-(-)-epicatechin 7-*O*-glucuronide	C_22_H_24_O_12_	76.365	[M + H]^+^	480.1268	481.1341	481.1340	0.19	Hops
	**Flavanones**								
66	Eriocitrin	C_27_H_32_O_15_	21.939	[M − H]^−^	596.1741	595.1668	595.1668	0.00	Hops
67	Naringenin 7-*O*-glucoside	C_21_H_22_O_10_	37.278	[M − H]^−^	434.1213	433.1140	433.1121	−1.57	* Hops, juniper berries
68	Hesperetin 3’-*O*-glucuronide	C_22_H_22_O_12_	48.476	[M − H]^−^	478.1111	477.1038	477.1051	2.88	* Hops, juniper berries
	**Flavones**								
69	Apigenin 7-*O*-glucuronide	C_21_H_18_O_11_	8.564	[M + H]^+^	446.0849	447.0922	447.0908	−0.89	Hops
70	Isorhoifolin	C_27_H_30_O_14_	16.539	[M + H]^+^	578.1636	579.1709	579.1675	−5.75	Juniper berries
71	Apigenin 6,8-di-C-glucoside	C_27_H_30_O_15_	42.794	[M − H]^−^	594.1585	593.1512	593.1532	3.11	* Hops, juniper berries
72	Chrysoeriol 7-*O*-(6’’-malonyl-apiosyl-glucoside)	C_30_H_32_O_18_	43.739	[M − H]^−^	680.1589	679.1516	679.1521	1.15	Hops
73	6-Hydroxyluteolin 7-*O*-rhamnoside	C_21_H_20_O_11_	45.627	[M − H]^−^	448.1006	447.0933	447.0949	3.41	* Hops, juniper berries
74	Apigenin 6-C-glucoside	C_21_H_20_O_10_	46.906	[M − H]^−^	432.1056	431.0983	431.0992	1.55	Juniper berries
75	Chrysoeriol 7-*O*-glucoside	C_22_H_22_O_11_	48.695	[M − H]^−^	462.1162	461.1089	461.1095	1.03	Juniper berries
76	Apigenin 7-*O*-apiosyl-glucoside	C_26_H_28_O_14_	55.335	[M + H]^+^	564.1479	565.1552	565.1538	−3.00	Juniper berries
77	Cirsilineol	C_18_H_16_O_7_	80.994	[M + H]^+^	344.0896	345.0969	345.0957	−2.40	Juniper berries
78	Gardenin B	C_19_H_18_O_7_	82.411	[M + H]^+^	358.1053	359.1126	359.1116	−2.73	Hops
	**Flavonols**								
79	Kaempferol 3-*O*-xylosyl-glucoside	C_26_H_28_O_15_	22.777	[M − H]^−^/*[M + H]^+^	580.1428	581.1501	581.1510	2.14	* Hops, juniper berries
80	Patuletin 3-*O*-glucosyl-(1->6)-[apiosyl(1->2)]-glucoside	C_33_H_40_O_22_	28.535	[M − H]^−^	788.2011	787.1938	787.1965	1.67	Juniper berries
81	Kaempferol 3,7,4’-*O*-triglucoside	C_33_H_40_O_21_	29.079	[M − H]^−^	772.2062	771.1989	771.1994	0.21	* Hops, juniper berries
82	Myricetin 3-*O*-rutinoside	C_27_H_30_O_17_	31.547	[M − H]^−^	626.1483	625.1410	625.1416	1.20	* Hops, juniper berries
83	Kaempferol 3-*O*-glucosyl-rhamnosyl-galactoside	C_33_H_40_O_20_	31.623	[M + H]^+^	756.2113	756.2059	757.2133	0.08	* Hops, juniper berries
84	Myricetin 3-*O*-glucoside	C_21_H_20_O_13_	33.220	[M − H]^−^	480.0904	479.0831	479.0859	7.56	* Hops, juniper berries
85	Myricetin	C_15_H_10_O_8_	33.345	[M + H]^+^	318.0376	319.0449	319.0427	−5.24	* Hops, juniper berries
86	Quercetin 3-*O*-xylosyl-rutinoside	C_32_H_38_O_20_	33.419	[M − H]^−^	742.1956	741.1883	741.1900	2.02	Hops
87	Quercetin 3’-*O*-glucuronide	C_21_H_18_O_13_	34.131	[M + H]^+^	478.0747	479.0820	479.0810	−1.82	Juniper berries
88	Kaempferol 3,7-*O*-diglucoside	C_27_H_30_O_16_	34.512	[M − H]^−^	610.1534	609.1461	609.1495	5.53	* Hops, juniper berries
89	Kaempferol 3-*O*-(2’’-rhamnosyl-galactoside) 7-*O*-rhamnoside	C_33_H_40_O_19_	34.644	[M − H]^−^	740.2164	739.2091	739.2125	4.28	* Hops, juniper berries
90	Quercetin 3-*O*-glucosyl-xyloside	C_26_H_28_O_16_	35.920	* [M − H]^−^/[M + H]^+^	596.1377	595.1304	595.1328	4.33	* Hops, juniper berries
91	Myricetin 3-*O*-arabinoside	C_20_H_18_O_12_	37.063	[M + H]^+^	450.0798	451.0871	451.0850	−4.61	Juniper berries
92	Spinacetin 3-*O*-glucosyl-(1->6)-glucoside	C_29_H_34_O_18_	38.027	[M − H]^−^	670.1745	669.1672	669.1689	1.98	Juniper berries
93	Myricetin 3-*O*-rhamnoside	C_21_H_20_O_12_	38.637	[M − H]^−^	464.0955	463.0882	463.0912	6.85	* Hops, juniper berries
94	Isorhamnetin 3-*O*-glucoside 7-*O*-rhamnoside	C_28_H_32_O_16_	38.762	[M − H]^−^/* [M + H]^+^	624.1690	625.1763	625.1772	0.78	* Hops, juniper berries
95	Quercetin 3-*O*-(6”-malonyl-glucoside)	C_24_H_22_O_15_	42.695	[M − H]^−^	550.0959	549.0886	549.0901	2.88	Hops
96	Quercetin 3-*O*-arabinoside	C_20_H_18_O_11_	43.599	[M − H]^−^/*[M + H]^+^	434.0849	435.0922	435.0925	−0.02	* Hops, juniper berries
97	Kaempferol 3-*O*-(6’’-acetyl-galactoside) 7-*O*-rhamnoside	C_29_H_32_O_16_	43.705	[M − H]^−^	636.1690	635.1617	635.1637	1.29	Hops
98	5,4’-Dihydroxy-3,3’-dimethoxy-6:7-methylenedioxyflavone 4’-*O*-glucuronide	C_24_H_22_O_14_	51.110	[M − H]^−^	534.1010	533.0937	533.0944	1.52	Hops
99	Isorhamnetin	C_16_H_12_O_7_	53.313	[M − H]^−^	316.0583	315.0510	315.0508	0.56	* Hops, juniper berries
	**Isoflavonoids**								
100	6’’-*O*-Acetylgenistin	C_23_H_22_O_11_	10.791	[M + H]^+^	474.1162	475.1235	475.1202	−6.50	Juniper berries
101	4’-Methoxy-2’,3,7-trihydroxyisoflavanone	C_16_H_14_O_6_	20.839	[M + H]^+^	302.0790	303.0863	303.0847	−4.47	Hops
102	6’’-*O*-Acetyldaidzin	C_23_H_22_O_10_	21.965	[M + H]^+^	458.1213	459.1286	459.1279	−0.27	Hops
103	3’-Hydroxydaidzein	C_15_H_10_O_5_	41.172	[M + H]^+^	270.0528	271.0601	271.0592	−3.12	Juniper berries
104	3’-Hydroxygenistein	C_15_H_10_O_6_	45.660	[M − H]^−^	286.0477	285.0404	285.0404	−0.09	* Hops, juniper berries
105	3’,4’,5,7-Tetrahydroxyisoflavanone	C_15_H_12_O_6_	50.083	[M − H]^−^	288.0634	287.0561	287.0576	5.34	* Hops, juniper berries
106	Irisolidone 7-*O*-glucuronide	C_23_H_22_O_12_	51.143	[M − H]^−^	490.1111	489.1038	489.1049	2.04	Hops
107	5,6,7,3’,4’-Pentahydroxyisoflavone	C_15_H_10_O_7_	69.083	[M − H]^−^	302.0427	301.0354	301.0375	7.30	* Hops, juniper berries
108	2’-Hydroxyformononetin	C_16_H_12_O_5_	74.940	[M + H]^+^	284.0685	285.0758	285.0766	2.04	Hops
109	2’,7-Dihydroxy-4’,5’-dimethoxyisoflavone	C_17_H_14_O_6_	78.145	[M + H]^+^	314.0790	315.0863	315.0846	−2.58	Juniper berries
110	3’-Hydroxymelanettin	C_16_H_12_O_6_	78.609	[M + H]^+^	300.0634	301.0707	301.0707	0.58	Juniper berries
111	Sativanone	C_17_H_16_O_5_	79.413	[M − H]^−^/*[M + H]^+^	300.0998	301.1071	301.1069	0.77	* Hops, juniper berries
112	Dihydrobiochanin A	C_16_H_14_O_5_	82.336	[M + H]^+^	286.0841	287.0914	287.0913	0.05	Juniper berries
**Lignans**									
113	Episesamin	C_20_H_18_O_6_	13.643	[M − H]^−^	354.1103	353.1030	353.1019	−4.36	Juniper berries
114	Secoisolariciresinol	C_20_H_26_O_6_	46.713	[M + H]^+^	362.1729	363.1802	363.1780	−5.44	Hops
115	Anhydro-secoisolariciresinol	C_20_H_24_O_5_	46.747	[M + H]^+^	344.1624	345.1697	345.1678	−5.38	Hops
116	7-Hydroxymatairesinol	C_20_H_22_O_7_	49.441	[M − H]^−^	374.1366	373.1293	373.1297	1.93	Juniper berries
117	Lariciresinol-sesquilignan	C_30_H_36_O_10_	52.522	[M − H]^−^	556.2308	555.2235	555.2231	−0.48	Juniper berries
118	Syringaresinol	C_22_H_26_O_8_	65.952	[M − H]^−^	418.1628	417.1555	417.1561	0.46	* Hops, juniper berries
119	Matairesinol	C_20_H_22_O_6_	77.250	[M + H]^+^	358.1416	359.1489	359.1470	−4.74	Juniper berries
120	Conidendrin	C_20_H_20_O_6_	77.756	[M + H]^+^	356.1260	357.1333	357.1344	2.21	Hops
**Stilbenes**									
121	Resveratrol	C_14_H_12_O_3_	38.282	[M + H]^+^	228.0786	229.0859	229.0871	4.74	Hops
122	Piceatannol 3-*O*-glucoside	C_20_H_22_O_9_	49.888	[M − H]^−^	406.1264	405.1191	405.1207	3.00	Juniper berries
123	4’-Hydroxy-3,4,5-trimethoxystilbene	C_17_H_18_O_4_	78.253	[M + H]^+^	286.1205	287.1278	287.1287	1.87	* Hops, juniper berries
**Hydroxybenzaldehydes**									
124	4-Hydroxybenzaldehyde	C_7_H_6_O_2_	26.826	[M − H]^−^	122.0368	121.0295	121.0306	9.07	Hops
**Other polyphenols**									
	**Alkylmethoxyphenols**								
125	4-Ethylguaiacol	C_9_H_12_O_2_	55.500	[M − H]^−^	152.0837	151.0764	151.0770	2.75	Hops
	**Alkylphenols**								
126	4-Ethylcatechol	C_8_H_10_O_2_	48.128	[M − H]^−^	138.0681	137.0608	137.0607	−0.35	Hops
	**Hydroxybenzaldehydes**								
127	*p*-Anisaldehyde	C_8_H_8_O_2_	12.662	* [M − H]^−^/[M + H]^+^	136.0524	135.0451	135.0456	4.06	* Hops, juniper berries
	**Hydroxybenzoketones**								
128	2,3-Dihydroxy-1-guaiacylpropanone	C_10_H_12_O_5_	13.126	* [M − H]^−^/[M + H]^+^	212.0685	211.0612	211.0622	6.08	* Hops, juniper berries
	**Hydroxycoumarins**								
129	4-Hydroxycoumarin	C_9_H_6_O_3_	12.589	[M + H]^+^	162.0317	163.0390	163.0375	−8.98	Hops
130	Coumarin	C_9_H_6_O_2_	17.642	[M + H]^+^	146.0368	147.0441	147.0429	−1.87	Hops
131	Mellein	C_10_H_10_O_3_	38.100	[M − H]^−^/*[M + H]^+^	178.0630	179.0703	179.0688	−6.79	* Hops, juniper berries
132	Scopoletin	C_10_H_8_O_4_	56.063	[M − H]^−^	192.0423	191.0350	191.0350	0.49	Hops
133	Esculetin	C_9_H_6_O_4_	82.958	[M + H]^+^	178.0266	179.0339	179.0332	−2.96	Hops
	**Hydroxyphenylpropenes**								
134	Anethole	C_10_H_12_O	31.126	[M + H]^+^	148.0888	149.0961	149.0950	−7.12	Hops
135	Acetyl eugenol	C_12_H_14_O_3_	80.666	[M − H]^−^	206.0943	205.0870	205.0883	3.91	Juniper berries
	**Other polyphenols**								
136	Arbutin	C_12_H_16_O_7_	6.785	[M − H]^−^	272.0896	271.0823	271.0836	2.33	Juniper berries
137	Pyrogallol	C_6_H_6_O_3_	6.957	[M + H]^+^	126.0317	127.0390	127.0391	0.29	* Hops, juniper berries
138	Catechol	C_6_H_6_O_2_	12.335	[M − H]^−^	110.0368	109.0295	109.0305	9.02	Juniper berries
139	3,4-Dihydroxyphenylglycol	C_8_H_10_O_4_	13.010	[M − H]^−^	170.0579	169.0506	169.0503	−2.96	Hops
140	Salvianolic acid G	C_20_H_18_O_10_	49.457	[M − H]^−^	418.0900	417.0827	417.0831	0.75	Juniper berries
	**Tyrosols**								
141	Oleoside 11-methylester	C_17_H_24_O_11_	9.458	[M + H]^+^	404.1319	405.1392	405.1364	−1.45	Hops
142	Hydroxytyrosol 4-*O*-glucoside	C_14_H_20_O_8_	10.443	[M − H]^−^	316.1158	315.1085	315.1072	−4.83	Hops
143	3,4-DHPEA-EDA	C_17_H_20_O_6_	50.083	[M − H]^−^	320.1260	319.1187	319.1179	−3.03	Hops
144	*p*-HPEA-EDA	C_17_H_20_O_5_	50.133	[M − H]^−^	304.1311	303.1238	303.1254	4.55	Hops
145	3,4-DHPEA-AC	C_10_H_12_O_4_	54.589	[M − H]^−^	196.0736	195.0663	195.0667	1.86	* Hops, juniper berries
	**Phenolic terpenes**								
146	Thymol	C_10_H_14_O	29.593	[M + H]^+^	150.1045	151.1118	151.1108	−6.67	Juniper berries
147	Rosmanol	C_20_H_26_O_5_	80.307	[M + H]^+^	346.1780	347.1853	347.1841	−3.49	Hops
148	Carnosic acid	C_20_H_28_O_4_	84.191	[M − H]^−^	332.1988	331.1915	331.1935	4.86	Hops
**Non-phenolic metabolites**									
149	1,3,5-Trimethoxybenzene	C_9_H_12_O_3_	41.900	[M − H]^−^	168.0786	167.0713	167.0724	5.35	* Hops, juniper berries

* Data presented in the table are from the sample indicated with an asterisk “*”. Also, the compound showing both modes of ionization [M − H]^−^/[M + H]^+^), “*” mode of ionization belongs to the “*” sample.

**Table 3:** Quantification of polyphenolic compounds in hops and juniper berries samples by HPLC-PDA.

No.	Compounds Name	Chemical Formula	RT (min)	Standard Curve	Hops (mg/g _dw_)	Juniper (mg/g _dw_)	Polyphenol Class
1	Gallic acid	C_7_H_6_O_5_	6.836	y = 2531.9x + 12238	3.41 ± 0.02	-	Phenolic acids
2	Protocatechuic acid	C_7_H_6_O_4_	12.569	y = 1824x − 16182	2.25 ± 0.01 ^b^	11.46 ± 0.03 ^a^	Phenolic acids
3	Caftaric acid	C_13_H_12_O_9_	13.774	y = 3500.2x − 43822	0.72 ± 0.01	-	Phenolic acids
4	*p*-hydroxybenzoic acid	C_7_H_6_O_3_	20.24	y = 1387.5x + 5575.1	1.87 ± 0.01 ^b^	3.12 ± 0.01 ^a^	Phenolic acids
5	Cholrogenic acid	C_16_H_18_O_9_	20.579	y = 3043.6x + 4706.3	16.48 ± 0.03	-	Phenolic acids
6	Caffeic acid	C_9_H_8_O_4_	25.001	y = 5622.4x + 23944	0.11 ± 0.01 ^a^	0.14 ± 0.01 ^a^	Phenolic acids
7	Syringic acid	C_9_H_10_O_5_	26.739	y = 2900.6x + 65091	0.03 ± 0.01	-	Phenolic acids
8	Coumaric acid	C_9_H_8_O_3_	34.455	y = 6418.4x + 60121	-	0.32 ± 0.01	Phenolic acids
9	Catechin	C_15_H_14_O_6_	19.704	y = 779.41x + 2373.3	9.03 ± 0.02 ^a^	8.47 ± 0.02 ^b^	Flavonoids
10	Epicatechin gallate	C_22_H_18_O_10_	38.015	y = 22958x − 26657	0.02 ± 0.01	-	Flavonoids
11	Quercetin-3-*O*-galactoside	C_21_ H_20_ O_12_	40.134	y = 23472x + 185001	0.22 ± 0.01 ^b^	0.73 ± 0.01 ^a^	Flavonoids
12	Quercetin-3-*O*-glucuronide	C_21_H_18_O_13_	40.659	y = 20578x − 36888	0.15 ± 0.01 ^a^	0.07 ± 0.01 ^b^	Flavonoids
13	Kaempferol-3-*O*-glucoside	C_21_H_20_O_11_	47.111	y = 22405x − 33766	4.33 ± 0.02 ^a^	1.47 ± 0.02 ^b^	Flavonoids
14	Quercetin	C_15_H_10_O_7_	70.098	y = 2585.7x − 29267	1.03 ± 0.01 ^a^	0.74 ± 0.01 ^b^	Flavonoids
15	kaempferol	C_15_H_10_O_6_	80.347	y = 4425.8x − 110841	0.44 ± 0.01 ^b^	3.37 ± 0.01 ^a^	Flavonoids

All data are the mean ± SD of three replicates. Means followed by different letters (^a, b^) within the same column are significantly different (*p* < 0.05) from each other. Data of hops and juniper berries are reported on a dry weight basis.

## Supplementary Materials

The following are available online at https://www.mdpi.com/2304-8158/9/1/7/s1, Figure S1: LC-ESI-QTOF/MS basic peak chromatograph (BPC) for characterization of phenolic compounds of juniper berries and hops samples, Figure S2: Extracted ion chromatogram and their mass spectrum, Table S1: Phenolic compounds detected and tentatively characterised in hops extracts by using LC-ESI-QTOF/MS in both positive and negative ionisation modes, Table S2: Phenolic compounds detected and tentatively characterised in juniper berries extracts by using LC-ESI-QTOF/MS in both positive and negative ionisation modes.
