# Supplementary material for: LC-ESI-QTOF/MS Characterization of Phenolic Compounds from Medicinal Plants (Hops and Juniper Berries) and Their Antioxidant Activity
Source: Foods. 2019 Dec 20;9(1):7. doi: 10.3390/foods9010007 (PMC7023254; doi:10.3390/foods9010007)
Supplement: Supplementary file 1 [file foods-09-00007-s001.pdf]

Supplemental material

LC-ESI-Q-TOF/MS Characterization of phenolic compounds from medicinal plants (hops & juniper berries) and their antioxidant activity

Jiafei Tang, Frank R. Dunshea and Hafiz A.R. Suleria*

School of Agriculture and Food, Faculty of Veterinary and Agricultural Sciences, The University of Melbourne, Parkville, VIC 3010, Australia; jiafeit@student.unimelb.edu.au (J.T.); fdunshea@unimelb.edu.au (F.R.D)

* Correspondence: hafiz.suleria@unimelb.edu.au; Tel.: +61-470-439-670

Received: date; Accepted: date; Published: date

Abstract: Hops (*Humulus lupulus* L.) and juniper berries (*Juniperus communis* L.) are two important medicinal plants widely used in the food, beverage and pharmaceutical industries due to their strong antioxidant capacity, which is attributed to the presence of polyphenols. The present study was conducted to comprehensively characterize polyphenols from hops and juniper berries using the LC-ESI-QTOF/MS and to assess their antioxidant capacity. For antioxidant capacity, total phenolic content, flavonoid, tannins and three antioxidant assays including 2,2-diphenyl-1-picrylhydrazyl (DPPH) antioxidant assay, 2,2-azino-bis-3-ethylbenzothiazoline-6-sulfonic acid (ABTS) radical cation decolorization assay and ferric reducing-antioxidant power (FRAP) were measured. Hops presented the higher phenolic content (23.11 ± 0.03 mg/g_{dw}) which corresponded to its strong antioxidant activity as compared to the juniper berries. Using the LC-ESI-QTOF/MS, a total of 148 phenolic compounds were tentatively identified in juniper and hops, among which phenolic acids including (hydroxybenzoic acids, hydroxycinnamic acids and hydroxyphenylpropanoic acids) and flavonoids (mainly anthocyanins, flavones, flavonols, and isoflavonoids) were the main polyphenols, which may contribute to their antioxidant capacity. Furthermore, the HPLC quantitative analysis showed that both samples had high concentration of phenolic acids and flavonoids. In the HPLC quantification, the predominant phenolic acids in hops and juniper berries were chlorogenic acid (16.48 ± 0.03 mg/g_{dw}) and protocatechuic acid (11.46 ± 0.03 mg/g_{dw}), respectively. The obtained results highlight the importance of hops and juniper berries as a rich source of functional ingredients in different food, beverage and pharmaceutical industries.

Keywords: Medicinal plants; Hops; juniper berries; polyphenols; LC-ESI-QTOF/MS and antioxidant activities

Table (S1). Phenolic compounds detected and tentatively characterised in hops extracts by using LC-ESI-QTOF/MS in both positive and negative ionisation modes.

Peak No.	Proposed Compounds	Molecular Formula	Retention Time (min)	Mode of Ionization (ESI- / ESI+)	Molecular Weight	Theoretical (*m/z*)	Observed (*m/z*)	Mass Error (ppm)
**Phenolic acids**								
**Hydroxybenzoic acids**								
1	Galloyl glucose	$C_{13}H_{16}O_{10}$	6.583	ESI^{-} / $[M-H]^{-}$	332.0743	331.0670	331.0693	6.70
2	Gallic acid	$C_7H_6O_5$	6.749	ESI^{-} / $[M-H]^{-}$	170.0215	169.0142	169.0159	9.68
3	Protocatechuic acid 4-*O*-glucoside	$C_{13}H_{16}O_9$	9.151	ESI^{-} / $[M-H]^{-}$	316.0794	315.0721	315.0746	7.97
4	2,3-Dihydroxybenzoic acid	$C_7H_6O_4$	12.348	ESI^{-} / $[M-H]^{-}$	154.0266	153.0193	153.0203	6.14
5	2-Hydroxybenzoic acid	$C_7H_6O_3$	19.935	ESI^{-} / $[M-H]^{-}$	138.0317	137.0244	137.0249	3.69
**Hydroxycinnamic acids**								
6	3-Caffeoylquinic acid	$C_{16}H_{18}O_9$	12.629	ESI^{-} / $[M-H]^{-}$	354.0951	353.0878	353.0894	1.84
7	3-Sinapoylquinic acid	$C_{18}H_{22}O_{10}$	13.815	ESI^{+} / $[M+H]^{+}$	398.1213	399.1286	399.1291	0.88
8	Caffeic acid 3-*O*-glucuronide	$C_{15}H_{16}O_{10}$	15.396	ESI^{-} / $[M-H]^{-}$	356.0743	355.0670	355.0680	3.79
9	3-*p*-Coumaroylquinic acid	$C_{16}H_{18}O_8$	17.665	ESI^{-} / $[M-H]^{-}$	338.1002	337.0929	337.0949	6.22
10	Rosmarinic acid	$C_{18}H_{16}O_8$	17.665	ESI^{-} / $[M-H]^{-}$	360.0845	359.0772	359.0780	8.44
11	*p*-Coumaric acid 4-*O*-glucoside	$C_{15}H_{18}O_8$	18.957	ESI^{-} / $[M-H]^{-}$	326.1002	325.0929	325.0920	-4.31
12	Ferulic acid 4-*O*-glucuronide	$C_{16}H_{18}O_{10}$	19.918	ESI^{-} / $[M-H]^{-}$	370.0900	369.0827	369.0833	1.54
13	3-Feruloylquinic acid	$C_{17}H_{20}O_9$	20.481	ESI^{-} / $[M-H]^{-}$	368.1107	367.1034	367.1038	0.94
14	Cinnamic acid	$C_9H_8O_2$	20.491	ESI^{+} / $[M+H]^{+}$	148.0524	149.0597	149.0587	-6.81
15	Ferulic acid 4-*O*-glucoside	$C_{16}H_{20}O_9$	22.916	ESI^{-} / $[M-H]^{-}$	356.1107	355.1034	355.1058	6.08
16	Caffeoyl glucose	$C_{15}H_{18}O_9$	24.076	ESI^{-} / $[M-H]^{-}$	342.0951	341.0878	341.0898	6.09
17	*p*-Coumaroyl tyrosine	$C_{18}H_{17}NO_5$	27.637	ESI^{-} / $[M-H]^{-}$	327.1107	326.1034	326.1042	-3.98
18	1,2-Disinapoylgentiobiose	$C_{34}H_{42}O_{19}$	37.991	ESI^{-} / $[M-H]^{-}$	754.2320	753.2247	753.2281	-5.47
19	*p*-Coumaric acid ethyl ester	$C_{11}H_{12}O_3$	81.109	ESI^{-} / $[M-H]^{-}$	192.0786	191.0713	191.0733	9.96

20	Isoferulic acid	$C_{10}H_{10}O_4$	81.881	ESI^+ / $[M+H]^+$	194.0579	195.0652	195.0656	2.46
**Hydroxyphenylacetic acids**								
21	2-Hydroxy-2-phenylacetic acid	$C_8H_8O_3$	15.247	ESI^- / $[M-H]^-$	152.0473	151.0400	151.0408	6.70
22	3,4-Dihydroxyphenylacetic acid	$C_8H_8O_4$	40.227	ESI^- / $[M-H]^-$	168.0423	167.0350	167.0367	9.63
**Hydroxyphenylpentanoic acids**								
23	3-Hydroxyphenylvaleric acid	$C_{11}H_{14}O_3$	8.978	ESI^+ / $[M+H]^+$	194.0943	195.1016	195.1019	-0.78
24	5-(3',4'-dihydroxyphenyl)-valeric acid	$C_{11}H_{14}O_4$	48.883	ESI^+ / $[M+H]^+$	210.0892	211.0965	211.0956	-4.05
25	5-(3',4',-dihydroxyphenyl)-γ-valerolactone	$C_{11}H_{12}O_4$	68.437	ESI^- / $[M-H]^-$	208.0736	207.0663	207.0679	8.32
**Hydroxyphenylpropanoic acids**								
26	Dihydrocaffeic acid 3-*O*-glucuronide	$C_{15}H_{18}O_{10}$	13.772	ESI^- / $[M-H]^-$	358.0900	357.0827	357.0831	1.32
27	Dihydrosinapic acid	$C_{11}H_{14}O_5$	15.909	ESI^- / $[M-H]^-$	226.0841	225.0768	225.0760	-5.07
28	Dihydroferulic acid 4-*O*-glucuronide	$C_{16}H_{20}O_{10}$	18.957	ESI^- / $[M-H]^-$	372.1056	371.0983	371.0962	-4.96
29	3-Hydroxy-3-(3-hydroxyphenyl) propionic acid	$C_9H_{10}O_4$	48.095	ESI^- / $[M-H]^-$	182.0579	181.0506	181.0524	9.61
30	3-Hydroxyphenylpropionic acid	$C_9H_{10}O_3$	49.139	ESI^- / $[M-H]^-$	166.0630	165.0557	165.0569	6.63
**Flavonoids**								
**Anthocyanins**								
31	Cyanidin 3-*O*-(6''-*p*-coumaroyl-glucoside)	$C_{30}H_{27}O_{13}$	8.140	ESI^- / $[M-H]^-$	595.1452	594.1379	594.1361	-2.65
32	Delphinidin 3-*O*-glucosyl-glucoside	$C_{27}H_{31}O_{17}$	32.143	ESI^- / $[M-H]^-$	627.1561	626.1488	626.1464	-4.36
33	Peonidin 3-*O*-sambubioside-5-*O*-glucoside	$C_{33}H_{41}O_{20}$	32.640	ESI^- / $[M-H]^-$	757.2191	756.2118	756.2098	-2.92
34	Cyanidin 3-*O*-sambubioside 5-*O*-glucoside	$C_{32}H_{39}O_{20}$	33.104	ESI^- / $[M-H]^-$	743.2035	742.1962	742.1933	-2.55
35	Pelargonidin 3-*O*-glucosyl-rutinoside	$C_{33}H_{41}O_{19}$	34.644	ESI^- / $[M-H]^-$	741.2242	740.2169	740.2153	-2.68
36	Delphinidin 3-*O*-sambubioside	$C_{26}H_{29}O_{16}$	35.903	ESI^- / $[M-H]^-$	597.1456	596.1383	596.1363	-2.73
37	Cyanidin 3,5-*O*-diglucoside	$C_{27}H_{31}O_{16}$	37.079	ESI^- / $[M-H]^-$	611.1612	610.1539	610.1530	-2.01
38	Cyanidin 3-*O*-(6''-malonyl-3''-glucosyl-glucoside)	$C_{30}H_{33}O_{19}$	38.189	ESI^- / $[M-H]^-$	697.1616	696.1543	696.1524	-2.24
39	Cyanidin 3-*O*-rutinoside	$C_{27}H_{31}O_{15}$	38.355	ESI^- / $[M-H]^-$	595.1663	594.1590	594.1570	-3.45

40	Delphinidin 3-*O*-glucoside	$C_{21}H_{21}O_{12}$	39.382	ESI^- / $[M-H]^-$	465.1033	464.0960	464.0945	-3.80
41	Peonidin 3-*O*-sophoroside	$C_{28}H_{33}O_{16}$	41.005	ESI^- / $[M-H]^-$	625.1769	624.1696	624.1682	-1.55
42	Delphinidin 3-*O*-(6"-acetyl-glucoside)	$C_{23}H_{23}O_{13}$	42.678	ESI^- / $[M-H]^-$	507.1139	506.1066	506.1040	-5.07
43	Pelargonidin 3,5-*O*-diglucoside	$C_{27}H_{31}C_{l}O_{15}$	42.844	ESI^- / $[M-H]^-$	630.1351	629.1278	629.1293	-0.28
44	Cyanidin 3-*O*-galactoside	$C_{21}H_{21}O_{11}$	43.275	ESI^- / $[M-H]^-$	449.1084	448.1011	448.0982	-6.42
45	Cyanidin 3-*O*-(6"-acetyl-glucoside)	$C_{23}H_{23}O_{12}$	51.143	ESI^- / $[M-H]^-$	491.1190	490.1117	490.1083	-5.46
46	Cyanidin	$C_{15}H_{11}O_{6}$	79.801	ESI^- / $[M-H]^-$	287.0556	286.0483	286.0468	-3.14
**Chalcones**								
47	Xanthohumol	$C_{21}H_{22}O_{5}$	82.941	ESI^+ / $[M+H]^+$	354.1467	355.1540	355.1523	-3.79
**Dihydrochalcones**								
48	3-Hydroxyphloretin 2'-*O*-glucoside	$C_{21}H_{24}O_{11}$	18.924	ESI^- / $[M-H]^-$	452.1319	451.1246	451.1252	-1.17
**Dihydroflavonols**								
49	Dihydroquercetin 3-*O*-rhamnoside	$C_{21}H_{22}O_{11}$	26.544	ESI^- / $[M-H]^-$	450.1162	449.1089	449.1103	3.17
50	Dihydromyricetin 3-*O*-rhamnoside	$C_{21}H_{22}O_{12}$	64.802	ESI^+ / $[M+H]^+$	466.1111	467.1184	467.1164	-3.04
**Flavanols**								
51	Procyanidin dimer B1	$C_{30}H_{26}O_{12}$	14.932	ESI^- / $[M-H]^-$	578.1424	577.1351	577.1355	0.25
52	(-)-Epigallocatechin	$C_{15}H_{14}O_{7}$	16.605	ESI^- / $[M-H]^-$	306.0740	305.0667	305.0668	0.88
53	Procyanidin trimer C1	$C_{45}H_{38}O_{18}$	18.576	ESI^- / $[M-H]^-$	866.2058	865.1985	865.1966	-2.37
54	4'-*O*-Methylepigallocatechin	$C_{16}H_{16}O_{7}$	24.450	ESI^+ / $[M+H]^+$	320.0896	321.0969	321.0959	-3.17
55	(-)-Epicatechin	$C_{15}H_{14}O_{6}$	25.848	ESI^- / $[M-H]^-$	290.0790	289.0717	289.0736	6.06
56	4''-*O*-Methylepigallocatechin 3-*O*-gallate	$C_{23}H_{20}O_{11}$	26.636	ESI^+ / $[M+H]^+$	472.1006	473.1079	473.1062	-3.01
57	Cinnamtannin A2	$C_{60}H_{50}O_{24}$	29.592	ESI^- / $[M-H]^-$	1154.2690	1153.2620	1153.2610	-0.97
58	3'-*O*-Methyl-(-)-epicatechin 7-*O*-glucuronide	$C_{22}H_{24}O_{12}$	76.365	ESI^+ / $[M+H]^+$	480.1268	481.1341	481.1340	0.19
**Flavanones**								
59	Eriocitrin	$C_{27}H_{32}O_{15}$	21.939	ESI^- / $[M-H]^-$	596.1741	595.1668	595.1668	0.00

60	Naringenin 7-*O*-glucoside	$C_{21}H_{22}O_{10}$	37.278	ESI^- / $[M-H]^-$	434.1213	433.1140	433.1121	-1.57
61	Hesperetin 3'-*O*-glucuronide	$C_{22}H_{22}O_{12}$	48.476	ESI^- / $[M-H]^-$	478.1111	477.1038	477.1051	2.88
**Flavones**								
62	Apigenin 7-*O*-glucuronide	$C_{21}H_{18}O_{11}$	8.564	ESI^+ / $[M+H]^+$	446.0849	447.0922	447.0908	-0.89
63	Apigenin 6,8-di-C-glucoside	$C_{27}H_{30}O_{15}$	42.794	ESI^- / $[M-H]^-$	594.1585	593.1512	593.1532	3.11
64	Chrysoeriol 7-*O*-(6''-malonyl-apiosyl-glucoside)	$C_{30}H_{32}O_{18}$	43.739	ESI^- / $[M-H]^-$	680.1589	679.1516	679.1521	1.15
65	6-Hydroxyluteolin 7-*O*-rhamnoside	$C_{21}H_{20}O_{11}$	45.627	ESI^- / $[M-H]^-$	448.1006	447.0933	447.0949	3.41
66	Gardenin B	$C_{19}H_{18}O_{7}$	82.411	ESI^+ / $[M+H]^+$	358.1053	359.1126	359.1116	-2.73
**Flavonols**								
67	Kaempferol 3-*O*-xylosyl-glucoside	$C_{26}H_{28}O_{15}$	22.777	ESI^+ / $[M+H]^+$	580.1428	581.1501	581.1510	2.14
68	Kaempferol 3,7,4'-*O*-triglucoside	$C_{33}H_{40}O_{21}$	29.079	ESI^- / $[M-H]^-$	772.2062	771.1989	771.1994	0.21
69	Kaempferol 3-*O*-glucosyl-rhamnosyl-galactoside	$C_{33}H_{40}O_{20}$	31.514	ESI^- / $[M-H]^-$	756.2113	755.204	755.2043	0.08
70	Myricetin 3-*O*-rutinoside	$C_{27}H_{30}O_{17}$	31.547	ESI^- / $[M-H]^-$	626.1483	625.1410	625.1416	1.20
71	Myricetin 3-*O*-glucoside	$C_{21}H_{20}O_{13}$	33.220	ESI^- / $[M-H]^-$	480.0904	479.0831	479.0859	7.56
72	Myricetin	$C_{15}H_{10}O_{8}$	33.345	ESI^+ / $[M+H]^+$	318.0376	319.0449	319.0427	-5.24
73	Quercetin 3-*O*-xylosyl-rutinoside	$C_{32}H_{38}O_{20}$	33.419	ESI^- / $[M-H]^-$	742.1956	741.1883	741.1900	2.02
74	Kaempferol 3,7-*O*-diglucoside	$C_{27}H_{30}O_{16}$	34.512	ESI^- / $[M-H]^-$	610.1534	609.1461	609.1495	5.53
75	Kaempferol 3-*O*-(2''-rhamnosyl-galactoside) 7-*O*-rhamnoside	$C_{33}H_{40}O_{19}$	34.644	ESI^- / $[M-H]^-$	740.2164	739.2091	739.2125	4.28
76	Quercetin 3-*O*-glucosyl-xyloside	$C_{26}H_{28}O_{16}$	35.920	ESI^- / $[M-H]^-$	596.1377	595.1304	595.1328	4.33
77	Myricetin 3-*O*-rhamnoside	$C_{21}H_{20}O_{12}$	38.637	ESI^- / $[M-H]^-$	464.0955	463.0882	463.0912	6.85
78	Isorhamnetin 3-*O*-glucoside 7-*O*-rhamnoside	$C_{28}H_{32}O_{16}$	38.762	ESI^+ / $[M+H]^+$	624.1690	625.1763	625.1772	0.78
79	Quercetin 3-*O*-(6''-malonyl-glucoside)	$C_{24}H_{22}O_{15}$	42.695	ESI^- / $[M-H]^-$	550.0959	549.0886	549.0901	2.88
80	Quercetin 3-*O*-arabinoside	$C_{20}H_{18}O_{11}$	43.599	ESI^+ / $[M+H]^+$	434.0849	435.0922	435.0925	-0.02

	81	Kaempferol 3-*O*-(6''-acetyl-galactoside) 7-*O*-rhamnoside	$C_{29}H_{32}O_{16}$	43.705	ESI^- / $[M-H]^-$	636.1690	635.1617	635.1637	1.29
	82	5,4'-Dihydroxy-3,3'-dimethoxy-6:7-methylenedioxyflavone 4'-*O*-glucuronide	$C_{24}H_{22}O_{14}$	51.110	ESI^- / $[M-H]^-$	534.1010	533.0937	533.0944	1.52
	83	Isorhamnetin	$C_{16}H_{12}O_7$	53.313	ESI^- / $[M-H]^-$	316.0583	315.0510	315.0508	0.56
**Isoflavonoids**									
	84	4'-Methoxy-2',3,7-trihydroxyisoflavanone	$C_{16}H_{14}O_6$	20.839	ESI^+ / $[M+H]^+$	302.0790	303.0863	303.0847	-4.47
	85	6''-*O*-Acetyldaidzin	$C_{23}H_{22}O_{10}$	21.965	ESI^+ / $[M+H]^+$	458.1213	459.1286	459.1279	-0.27
	86	3'-Hydroxygenistein	$C_{15}H_{10}O_6$	45.660	ESI^- / $[M-H]^-$	286.0477	285.0404	285.0404	-0.09
	87	3',4',5,7-Tetrahydroxyisoflavanone	$C_{15}H_{12}O_6$	50.083	ESI^- / $[M-H]^-$	288.0634	287.0561	287.0576	5.34
	88	Irisolidone 7-*O*-glucuronide	$C_{23}H_{22}O_{12}$	51.143	ESI^- / $[M-H]^-$	490.1111	489.1038	489.1049	2.04
	89	5,6,7,3',4'-Pentahydroxyisoflavone	$C_{15}H_{10}O_7$	69.083	ESI^- / $[M-H]^-$	302.0427	301.0354	301.0375	7.30
	90	2'-Hydroxyformononetin	$C_{16}H_{12}O_5$	74.940	ESI^+ / $[M+H]^+$	284.0685	285.0758	285.0766	2.04
	91	Sativanone	$C_{17}H_{16}O_5$	79.413	ESI^+ / $[M+H]^+$	300.0998	301.1071	301.1069	0.77
**Lignans**									
	92	Secoisolariciresinol	$C_{20}H_{26}O_6$	46.713	ESI^+ / $[M+H]^+$	362.1729	363.1802	363.1780	-5.44
	93	Anhydro-secoisolariciresinol	$C_{20}H_{24}O_5$	46.747	ESI^+ / $[M+H]^+$	344.1624	345.1697	345.1678	-5.38
	94	Syringaresinol	$C_{22}H_{26}O_8$	65.952	ESI^- / $[M-H]^-$	418.1628	417.1555	417.1561	0.46
	95	Conidendrin	$C_{20}H_{20}O_6$	77.756	ESI^+ / $[M+H]^+$	356.1260	357.1333	357.1344	2.21
**Stilbenes**									
	96	Resveratrol	$C_{14}H_{12}O_3$	38.282	ESI^+ / $[M+H]^+$	228.0786	229.0859	229.0871	4.74
	97	4'-Hydroxy-3,4,5-trimethoxystilbene	$C_{17}H_{18}O_4$	78.253	ESI^+ / $[M+H]^+$	286.1205	287.1278	287.1287	1.87
**Hydroxybenzaldehydes**									
	98	4-Hydroxybenzaldehyde	$C_7H_6O_2$	26.826	ESI^- / $[M-H]^-$	122.0368	121.0295	121.0306	9.07
**Other polyphenols**									
**Alkylmethoxyphenols**									

99	4-Ethylguaiacol	$C_9H_{12}O_2$	55.500	ESI$^-$ / [M-H]$^-$	152.0837	151.0764	151.0770	2.75
**Alkylphenols**								
100	4-Ethylcatechol	$C_8H_{10}O_2$	48.128	ESI$^-$ / [M-H]$^-$	138.0681	137.0608	137.0607	-0.35
**Hydroxybenzaldehydes**								
101	p-Anisaldehyde	$C_8H_8O_2$	12.662	ESI$^-$ / [M-H]$^-$	136.0524	135.0451	135.0456	4.06
**Hydroxybenzoketones**								
102	2,3-Dihydroxy-1-guaiacylpropanone	$C_{10}H_{12}O_5$	13.126	ESI$^-$ / [M-H]$^-$	212.0685	211.0612	211.0622	6.08
**Hydroxycoumarins**								
103	4-Hydroxycoumarin	$C_9H_6O_3$	12.589	ESI$^+$ / [M+H]$^+$	162.0317	163.0390	163.0375	-8.98
104	Coumarin	$C_9H_6O_2$	17.642	ESI$^+$ / [M+H]$^+$	146.0368	147.0441	147.0429	-1.87
105	Mellein	$C_{10}H_{10}O_3$	38.100	ESI$^+$ / [M+H]$^+$	178.0630	179.0703	179.0688	-6.79
106	Scopoletin	$C_{10}H_8O_4$	56.063	ESI$^-$ / [M-H]$^-$	192.0423	191.0350	191.0350	0.49
107	Esculetin	$C_9H_6O_4$	82.958	ESI$^+$ / [M+H]$^+$	178.0266	179.0339	179.0332	-2.96
**Hydroxyphenylpropenes**								
108	Anethole	$C_{10}H_{12}O$	31.126	ESI$^+$ / [M+H]$^+$	148.0888	149.0961	149.0950	-7.12
**Other polyphenols**								
109	Pyrogallol	$C_6H_6O_3$	6.957	ESI$^+$ / [M+H]$^+$	126.0317	127.0390	127.0391	0.29
110	3,4-Dihydroxyphenylglycol	$C_8H_{10}O_4$	13.010	ESI$^-$ / [M-H]$^-$	170.0579	169.0506	169.0503	-2.96
**Phenolic terpenes**								
111	Rosmanol	$C_{20}H_{26}O_5$	80.307	ESI$^+$ / [M+H]$^+$	346.1780	347.1853	347.1841	-3.49
112	Carnosic acid	$C_{20}H_{28}O_4$	84.191	ESI$^-$ / [M-H]$^-$	332.1988	331.1915	331.1935	4.86
**Tyrosols**								
113	Oleoside 11-methylester	$C_{17}H_{24}O_{11}$	9.458	ESI$^+$ / [M+H]$^+$	404.1319	405.1392	405.1364	-1.45
114	Hydroxytyrosol 4-*O*-glucoside	$C_{14}H_{20}O_8$	10.443	ESI$^-$ / [M-H]$^-$	316.1158	315.1085	315.1072	-4.83
115	3,4-DHPEA-EDA	$C_{17}H_{20}O_6$	50.083	ESI$^-$ / [M-H]$^-$	320.1260	319.1187	319.1179	-3.03

116	p-HPEA-EDA	$C_{17}H_{20}O_5$	50.133	ESI^- / $[M-H]^-$	304.1311	303.1238	303.1254	4.55
117	3,4-DHPEA-AC	$C_{10}H_{12}O_4$	54.589	ESI^- / $[M-H]^-$	196.0736	195.0663	195.0667	1.86
**Non-phenolic metabolites**								
118	1,3,5-Trimethoxybenzene	$C_9H_{12}O_3$	41.900	ESI^- / $[M-H]^-$	168.0786	167.0713	167.0724	5.35

Table (S2). Phenolic compounds detected and tentatively characterised in juniper berries extracts by using LC-ESI-QTOF/MS in both positive and negative ionisation modes.

Peak No.	Proposed Compounds	Molecular Formula	Retention Time (min)	Mode of Ionization (ESI- / ESI+)	Molecular Weight	Theoretical (*m/z*)	Observed (*m/z*)	Mass Error (ppm)
**Phenolic acids**								
**Hydroxybenzoic acids**								
1	2-Hydroxybenzoic acid	$C_7H_6O_3$	8.193	ESI^- / $[M-H]^-$	138.0317	137.0244	137.0258	9.61
2	Protocatechuic acid 4-*O*-glucoside	$C_{13}H_{16}O_9$	11.440	ESI^- / $[M-H]^-$	316.0794	315.0721	315.0739	5.71
3	2,3-Dihydroxybenzoic acid	$C_7H_6O_4$	12.368	ESI^- / $[M-H]^-$	154.0266	153.0193	153.0204	6.83
4	4-*O*-Methylgallic acid	$C_8H_8O_5$	14.439	ESI^- / $[M-H]^-$	184.0372	183.0299	183.0306	4.71
5	Ellagic acid	$C_{14}H_6O_8$	45.283	ESI^- / $[M-H]^-$	302.0063	300.9990	300.9969	-7.08
**Hydroxycinnamic acids**								
6	3-Sinapoylquinic acid	$C_{18}H_{22}O_{10}$	8.078	ESI^- / $[M-H]^-$	398.1213	397.1140	397.1117	-6.42
7	*p*-Coumaroyl tyrosine	$C_{18}H_{17}NO_5$	8.456	ESI^+ / $[M+H]^+$	327.1107	328.1180	328.1172	-0.25
8	3-*p*-Coumaroylquinic acid	$C_{16}H_{18}O_8$	17.718	ESI^- / $[M-H]^-$	338.1002	337.0929	337.0955	8.10
9	Isoferulic acid	$C_{10}H_{10}O_4$	18.063	ESI^+ / $[M+H]^+$	194.0579	195.0652	195.0668	8.02
10	Cinnamic acid	$C_9H_8O_2$	41.635	ESI^+ / $[M+H]^+$	148.0524	149.0597	149.0591	-2.98
11	Verbascoside	$C_{29}H_{36}O_{15}$	54.046	ESI^- / $[M-H]^-$	624.2054	623.1981	623.1982	-0.85
12	*p*-Coumaric acid ethyl ester	$C_{11}H_{12}O_3$	81.080	ESI^- / $[M-H]^-$	192.0786	191.0713	191.0720	2.14
**Hydroxyphenylacetic acids**								
13	3,4-Dihydroxyphenylacetic acid	$C_8H_8O_4$	10.248	ESI^- / $[M-H]^-$	168.0423	167.0350	167.0363	8.00
14	2-Hydroxy-2-phenylacetic acid	$C_8H_8O_3$	14.223	ESI^- / $[M-H]^-$	152.0473	151.0400	151.0412	8.36
**Hydroxyphenylpentanoic acids**								
15	5-(3'-Methoxy-4'-hydroxyphenyl)-gamma -valerolactone	$C_{12}H_{14}O_4$	19.077	ESI^- / $[M-H]^-$	222.0892	221.0819	221.0835	7.82
16	5-(3',4',-dihydroxyphenyl)-gamma-valerolactone	$C_{11}H_{12}O_4$	55.537	ESI^- / $[M-H]^-$	208.0736	207.0663	207.0673	4.91
**Hydroxyphenylpropanoic acids**								

17	3-Hydroxyphenylpropionic acid	$C_9H_{10}O_3$	6.636	ESI^- / $[M-H]^-$	166.0630	165.0557	165.0555	-0.42
18	3-Hydroxy-3-(3-hydroxyphenyl) propionic acid	$C_9H_{10}O_4$	10.711	ESI^- / $[M-H]^-$	182.0579	181.0506	181.0524	8.43
**Flavonoids**								
**Anthocyanins**								
19	Delphinidin 3-*O*-glucosyl-glucoside	$C_{27}H_{31}O_{17}$	35.012	ESI^- / $[M-H]^-$	627.1561	626.1488	626.1469	-3.93
20	Delphinidin 3-*O*-glucoside	$C_{21}H_{21}O_{12}$	36.785	ESI^- / $[M-H]^-$	465.1033	464.0960	464.0953	-2.19
21	Cyanidin 3,5-*O*-diglucoside	$C_{27}H_{31}O_{16}$	37.100	ESI^- / $[M-H]^-$	611.1612	610.1539	610.1529	-2.14
22	Cyanidin 3-*O*-galactoside	$C_{21}H_{21}O_{11}$	39.104	ESI^- / $[M-H]^-$	449.1084	448.1011	448.0985	-6.36
23	Cyanidin 3-*O*-(6''-dioxalyl-glucoside)	$C_{25}H_{20}O_{17}$	45.432	ESI^- / $[M-H]^-$	592.0700	591.0627	591.0656	4.65
**Dihydrochalcones**								
24	3-Hydroxyphloretin 2'-*O*-glucoside	$C_{21}H_{24}O_{11}$	10.778	ESI^- / $[M-H]^-$	452.1319	451.1246	451.1268	4.95
25	Phloridzin	$C_{21}H_{24}O_{10}$	50.617	ESI^- / $[M-H]^-$	436.1369	435.1296	435.1301	1.77
**Flavanols**								
26	Procyanidin dimer B1	$C_{30}H_{26}O_{12}$	16.592	ESI^- / $[M-H]^-$	578.1424	577.1351	577.1370	4.42
27	Procyanidin trimer C1	$C_{45}H_{38}O_{18}$	17.404	ESI^- / $[M-H]^-$	866.2058	865.1985	865.2012	1.80
28	(-)-Epicatechin	$C_{15}H_{14}O_6$	25.849	ESI^+ / $[M+H]^+$	290.0790	291.0863	291.0849	-2.76
29	(+)-Gallocatechin 3-*O*-gallate	$C_{22}H_{18}O_{11}$	49.606	ESI^- / $[M-H]^-$	458.0849	457.0776	457.0769	0.42
**Flavanones**								
30	Naringenin 7-*O*-glucoside	$C_{21}H_{22}O_{10}$	34.963	ESI^- / $[M-H]^-$	434.1213	433.1140	433.1173	6.88
31	Hesperetin 3'-*O*-glucuronide	$C_{22}H_{22}O_{12}$	40.628	ESI^- / $[M-H]^-$	478.1111	477.1038	477.1052	2.88
**Flavones**								
32	Isorhoifolin	$C_{27}H_{30}O_{14}$	16.539	ESI^+ / $[M+H]^+$	578.1636	579.1709	579.1675	-5.75
33	6-Hydroxyluteolin 7-*O*-rhamnoside	$C_{21}H_{20}O_{11}$	44.090	ESI^- / $[M-H]^-$	448.1006	447.0933	447.0941	1.69
34	Apigenin 6-C-glucoside	$C_{21}H_{20}O_{10}$	46.906	ESI^- / $[M-H]^-$	432.1056	431.0983	431.0992	1.55
35	Apigenin 6,8-di-C-glucoside	$C_{27}H_{30}O_{15}$	48.364	ESI^- / $[M-H]^-$	594.1585	593.1512	593.1518	1.41

36	Chrysoeriol 7-*O*-glucoside	$C_{22}H_{22}O_{11}$	48.695	ESI^- / $[M-H]^-$	462.1162	461.1089	461.1095	1.03
37	Apigenin 7-*O*-apiosyl-glucoside	$C_{26}H_{28}O_{14}$	55.335	ESI^+ / $[M+H]^+$	564.1479	565.1552	565.1538	-3.00
38	Cirsilineol	$C_{18}H_{16}O_7$	80.994	ESI^+ / $[M+H]^+$	344.0896	345.0969	345.0957	-2.40
**Flavonols**								
39	Kaempferol 3,7,4'-*O*-triglucoside	$C_{33}H_{40}O_{21}$	21.611	ESI^- / $[M-H]^-$	772.2062	771.1989	771.2023	2.02
40	Patuletin 3-*O*-glucosyl-(1->6)-[apiosyl(1->2)]-glucoside	$C_{33}H_{40}O_{22}$	28.535	ESI^- / $[M-H]^-$	788.2011	787.1938	787.1965	1.67
41	Kaempferol 3-*O*-glucosyl-rhamnosyl-galactoside	$C_{33}H_{40}O_{20}$	31.583	ESI^- / $[M-H]^-$	756.2113	755.2040	755.2064	2.40
42	Myricetin 3-*O*-rutinoside	$C_{27}H_{30}O_{17}$	32.362	ESI^- / $[M-H]^-$	626.1483	625.1410	625.1437	3.97
43	Quercetin 3'-*O*-glucuronide	$C_{21}H_{18}O_{13}$	34.131	ESI^+ / $[M+H]^+$	478.0747	479.0820	479.0810	-1.82
44	Kaempferol 3-*O*-(2''-rhamnosyl-galactoside) 7-*O*-rhamnoside	$C_{33}H_{40}O_{19}$	34.648	ESI^- / $[M-H]^-$	740.2164	739.2091	739.2088	1.64
45	Kaempferol 3,7-*O*-diglucoside	$C_{27}H_{30}O_{16}$	36.073	ESI^- / $[M-H]^-$	610.1534	609.1461	609.1484	3.69
46	Myricetin	$C_{15}H_{10}O_8$	37.063	ESI^+ / $[M+H]^+$	318.0376	319.0449	319.0435	-4.57
47	Myricetin 3-*O*-arabinoside	$C_{20}H_{18}O_{12}$	37.063	ESI^+ / $[M+H]^+$	450.0798	451.0871	451.0850	-4.61
48	Spinacetin 3-*O*-glucosyl-(1->6)-glucoside	$C_{29}H_{34}O_{18}$	38.027	ESI^- / $[M-H]^-$	670.1745	669.1672	669.1689	1.98
49	Quercetin 3-*O*-glucosyl-xyloside	$C_{26}H_{28}O_{16}$	38.654	ESI^+ / $[M+H]^+$	596.1377	597.1450	597.1434	-3.25
50	Quercetin 3-*O*-arabinoside	$C_{20}H_{18}O_{11}$	42.102	ESI^- / $[M-H]^-$	434.0849	433.0776	433.0790	3.08
51	Myricetin 3-*O*-glucoside	$C_{21}H_{20}O_{13}$	44.819	ESI^- / $[M-H]^-$	480.0904	479.0831	479.0837	1.34
52	Kaempferol 3-*O*-xylosyl-glucoside	$C_{26}H_{28}O_{15}$	47.883	ESI^- / $[M-H]^-$	580.1428	579.1355	579.1373	2.55
53	Isorhamnetin 3-*O*-glucoside 7-*O*-rhamnoside	$C_{28}H_{32}O_{16}$	48.281	ESI^- / $[M-H]^-$	624.1690	623.1617	623.1618	0.69
54	Myricetin 3-*O*-rhamnoside	$C_{21}H_{20}O_{12}$	52.240	ESI^- / $[M-H]^-$	464.0955	463.0882	463.0889	1.61
55	Isorhamnetin	$C_{16}H_{12}O_7$	56.166	ESI^- / $[M-H]^-$	316.0583	315.0510	315.0520	3.02
**Isoflavonoids**								
56	6''-*O*-Acetylgenistin	$C_{23}H_{22}O_{11}$	10.791	ESI^+ / $[M+H]^+$	474.1162	475.1235	475.1202	-6.50
57	Sativanone	$C_{17}H_{16}O_5$	13.942	ESI^- / $[M-H]^-$	300.0998	299.0925	299.0931	-0.30
58	3'-Hydroxydaidzein	$C_{15}H_{10}O_5$	41.172	ESI^+ / $[M+H]^+$	270.0528	271.0601	271.0592	-3.12

	59	5,6,7,3',4'-Pentahydroxyisoflavone	$C_{15}H_{10}O_7$	45.366	ESI$^-$ / [M-H]$^-$	302.0427	301.0354	301.0364	3.33
	60	3',4',5,7-Tetrahydroxyisoflavanone	$C_{15}H_{12}O_6$	50.004	ESI$^-$ / [M-H]$^-$	288.0634	287.0561	287.0565	1.08
	61	3'-Hydroxygenistein	$C_{15}H_{10}O_6$	54.659	ESI$^-$ / [M-H]$^-$	286.0477	285.0404	285.0410	1.93
	62	2',7-Dihydroxy-4',5'-dimethoxyisoflavone	$C_{17}H_{14}O_6$	78.145	ESI$^+$ / [M+H]$^+$	314.0790	315.0863	315.0846	-2.58
	63	3'-Hydroxymelanettin	$C_{16}H_{12}O_6$	78.609	ESI$^+$ / [M+H]$^+$	300.0634	301.0707	301.0707	0.58
	64	Dihydrobiochanin A	$C_{16}H_{14}O_5$	82.336	ESI$^+$ / [M+H]$^+$	286.0841	287.0914	287.0913	0.05
**Lignans**									
	65	Episesamin	$C_{20}H_{18}O_6$	13.643	ESI$^-$ / [M-H]$^-$	354.1103	353.1030	353.1019	-4.36
	66	Syringaresinol	$C_{22}H_{26}O_8$	19.822	ESI$^-$ / [M-H]$^-$	418.1628	417.1555	417.1549	-1.70
	67	7-Hydroxymatairesinol	$C_{20}H_{22}O_7$	49.441	ESI$^-$ / [M-H]$^-$	374.1366	373.1293	373.1297	1.93
	68	Lariciresinol-sesquilignan	$C_{30}H_{36}O_{10}$	52.522	ESI$^-$ / [M-H]$^-$	556.2308	555.2235	555.2231	-0.48
	69	Matairesinol	$C_{20}H_{22}O_6$	77.250	ESI$^+$ / [M+H]$^+$	358.1416	359.1489	359.1470	-4.74
**Stilbenes**									
	70	Piceatannol 3-*O*-glucoside	$C_{20}H_{22}O_9$	49.888	ESI$^-$ / [M-H]$^-$	406.1264	405.1191	405.1207	3.00
	71	4'-Hydroxy-3,4,5-trimethoxystilbene	$C_{17}H_{18}O_4$	58.946	ESI$^+$ / [M+H]$^+$	286.1205	287.1278	287.1290	4.12
**Other polyphenols**									
**Hydroxybenzaldehydes**									
	72	*p*-Anisaldehyde	$C_8H_8O_2$	8.108	ESI$^+$ / [M+H]$^+$	136.0524	137.0597	137.0588	-6.03
**Hydroxybenzoketones**									
	73	2,3-Dihydroxy-1-guaiacylpropanone	$C_{10}H_{12}O_5$	11.454	ESI$^+$ / [M+H]$^+$	212.0685	213.0758	213.0755	2.59
**Hydroxycoumarins**									
	74	Mellein	$C_{10}H_{10}O_3$	45.597	ESI$^-$ / [M-H]$^-$	178.0630	177.0557	177.0568	6.44
**Hydroxyphenylpropenes**									
	75	Acetyl eugenol	$C_{12}H_{14}O_3$	80.666	ESI$^-$ / [M-H]$^-$	206.0943	205.0870	205.0883	3.91
**Other polyphenols**									

76	Arbutin	$C_{12}H_{16}O_7$	6.785	ESI^- / $[M-H]^-$	272.0896	271.0823	271.0836	2.33
77	Pyrogallol	$C_6H_6O_3$	9.565	ESI^+ / $[M+H]^+$	126.0317	127.0390	127.0383	-4.51
78	Catechol	$C_6H_6O_2$	12.335	ESI^- / $[M-H]^-$	110.0368	109.0295	109.0305	9.02
79	Salvianolic acid G	$C_{20}H_{18}O_{10}$	49.457	ESI^- / $[M-H]^-$	418.0900	417.0827	417.0831	0.75
**Phenolic terpenes**								
80	Thymol	$C_{10}H_{14}O$	29.593	ESI^+ / $[M+H]^+$	150.1045	151.1118	151.1108	-6.67
**Tyrosols**								
81	3,4-DHPEA-AC	$C_{10}H_{12}O_4$	6.653	ESI^- / $[M-H]^-$	196.0736	195.0663	195.0644	-9.35
**Non-phenolic metabolites**								
82	1,3,5-Trimethoxybenzene	$C_9H_{12}O_3$	7.282	ESI^- / $[M-H]^-$	168.0786	167.0713	167.0727	5.97

(a)

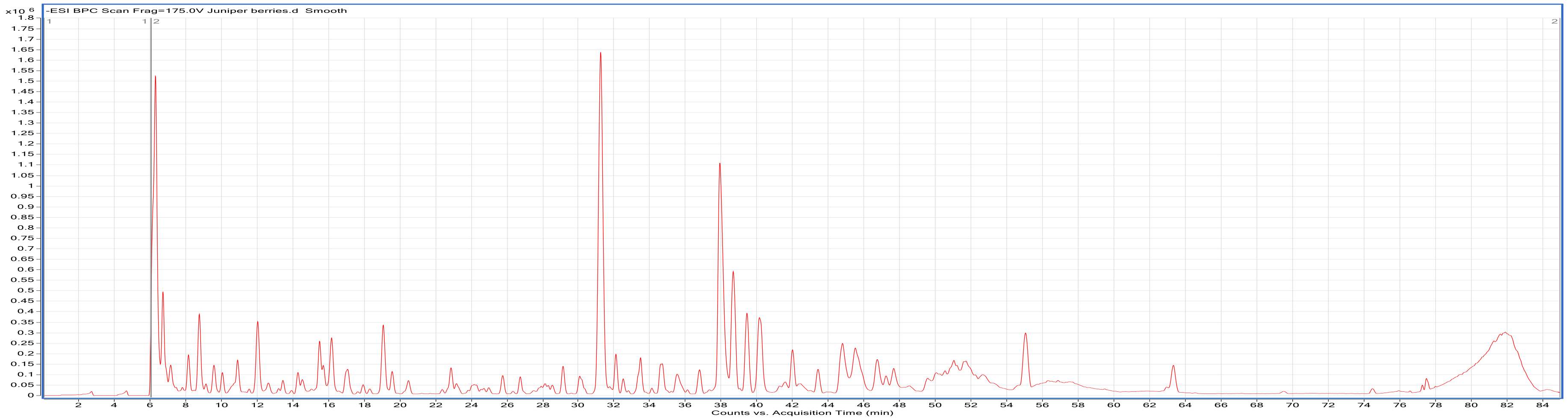
-ESI BPC Scan Frag=175.0V Juniper berries.d Smooth
x10 6
Counts vs. Acquisition Time (min)

(b)

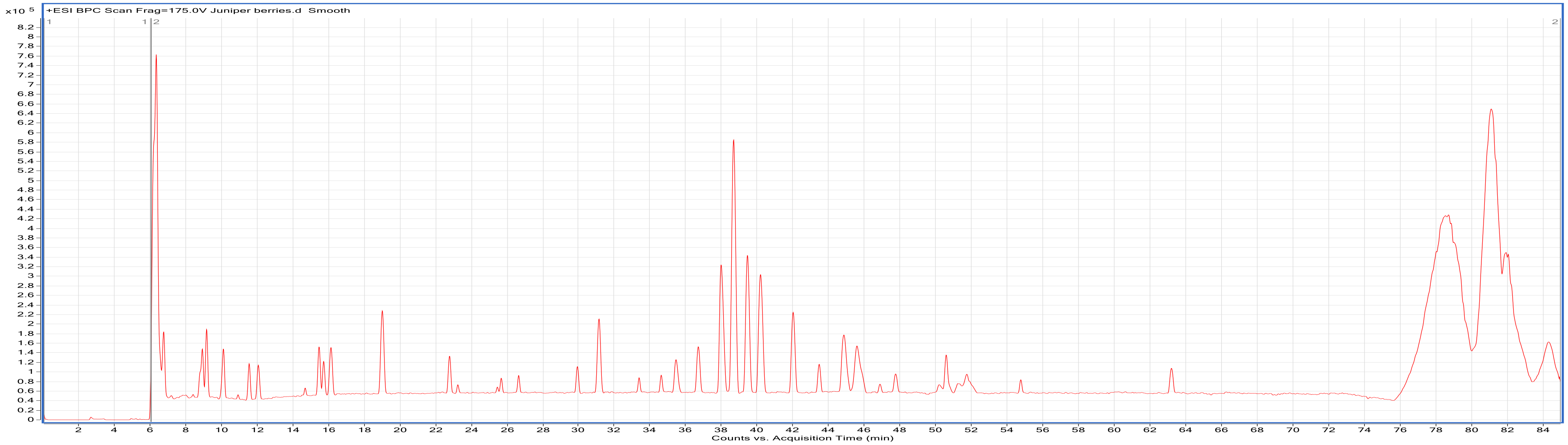
+ESI BPC Scan Frag=175.0V Juniper berries.d Smooth
x10 5
Counts vs. Acquisition Time (min)

(c)

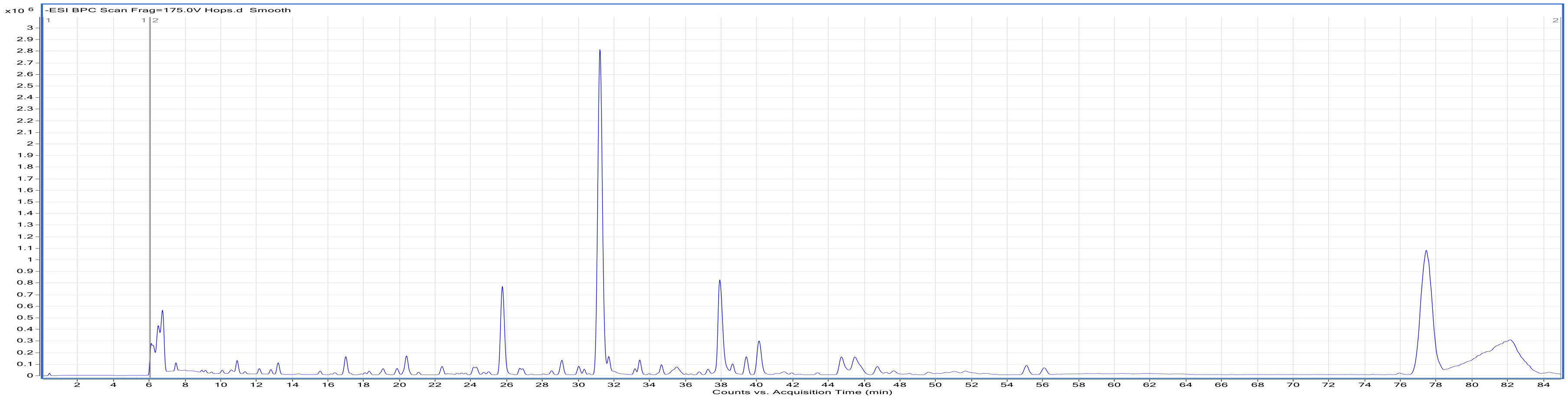

(d)

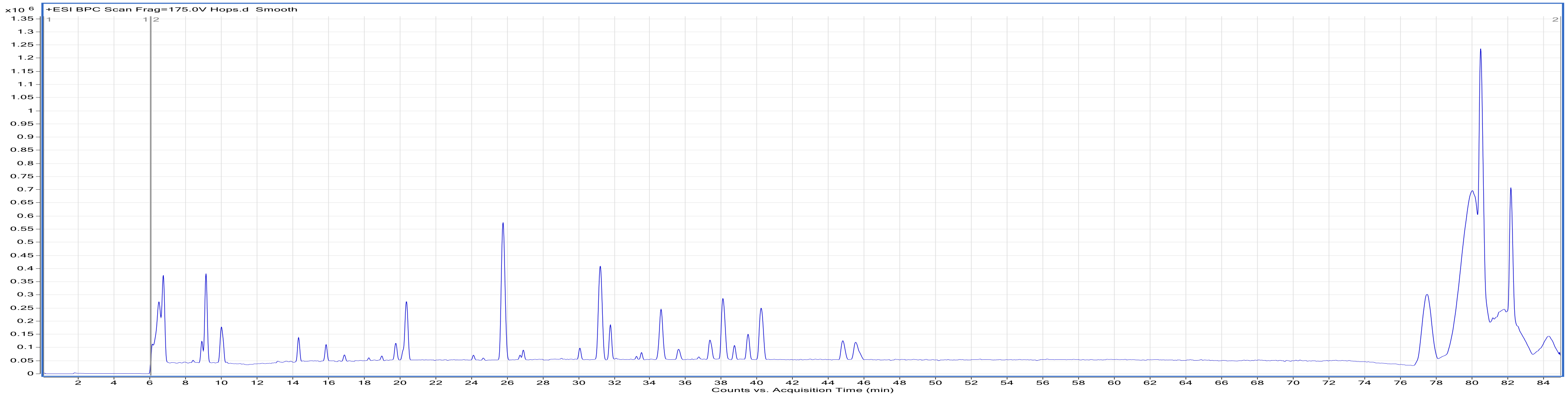

Figure (1S): LC-ESI-QTOF/MS basic peak chromatograph (BPC) for characterization of phenolic compounds of juniper berries and hops samples; **(a)** Juniper berries Base Peak Chromatogram (BPC) in negative ionization mode; **(b)** juniper berries BPC in positive ionization mode; **(c)** hops BPC in negative ionization mode; **(d)** hops BPC in positive ionization mode.

(a)

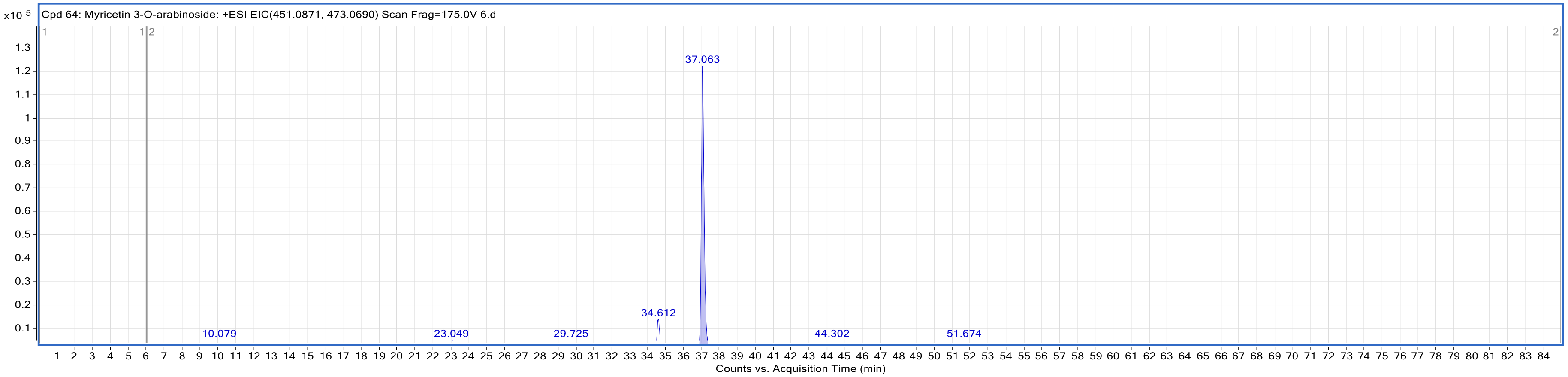

Cpd 64: Myricetin 3-O-arabinoside: +ESI EIC(451.0871, 473.0690) Scan Frag=175.0V 6.d
x10 5
37.063
34.612
10.079
23.049
29.725
44.302
51.674
Counts vs. Acquisition Time (min)

(b)

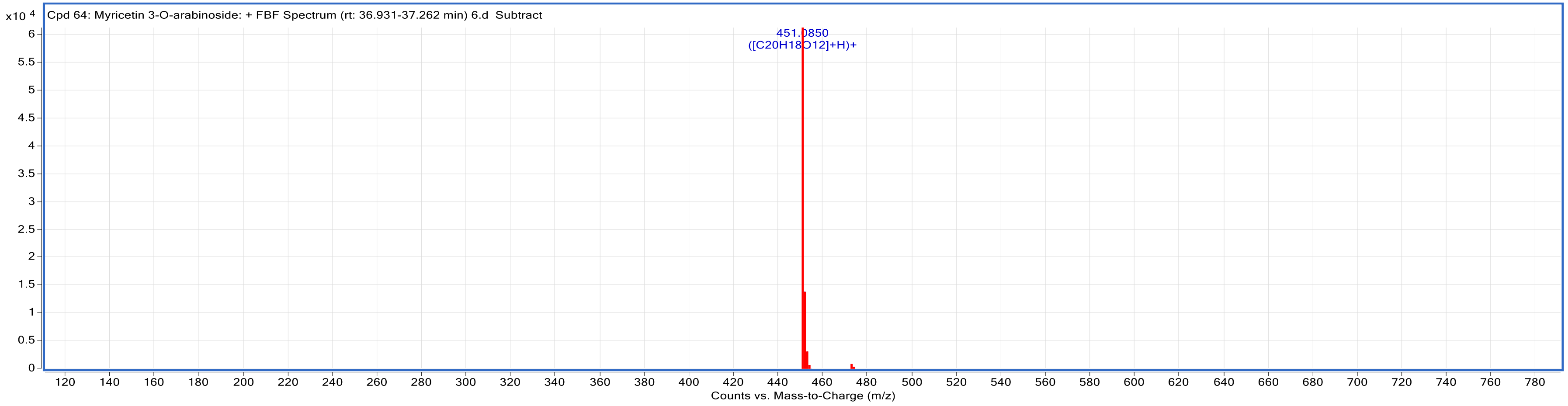

Cpd 64: Myricetin 3-O-arabinoside: + FBF Spectrum (rt: 36.931-37.262 min) 6.d Subtract
x10 4
451.0850
([C20H18O12]+H)+
Counts vs. Mass-to-Charge (m/z)

(c)

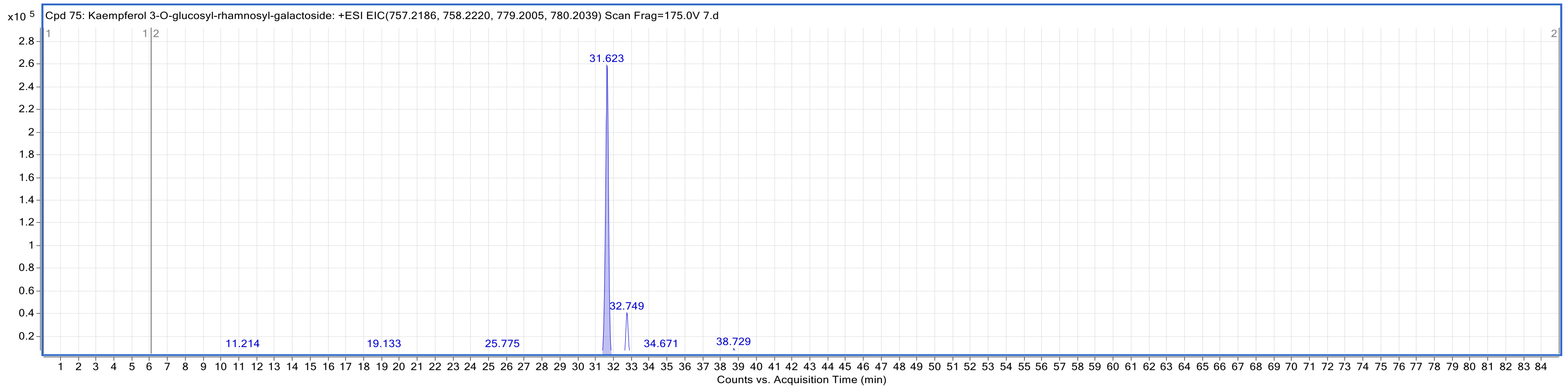
Cpd 75: Kaempferol 3-O-glucosyl-rhamnosyl-galactoside: +ESI EIC(757.2186, 758.2220, 779.2005, 780.2039) Scan Frag=175.0V 7.d
x10 5
31.623
32.749
11.214
19.133
25.775
34.671
38.729
Counts vs. Acquisition Time (min)

(d)

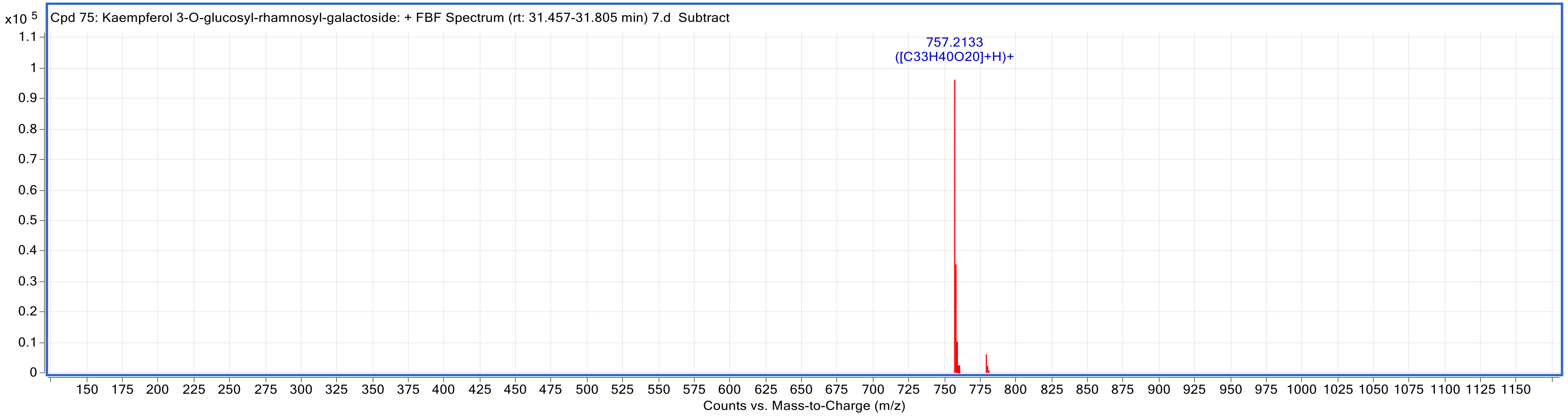
Cpd 75: Kaempferol 3-O-glucosyl-rhamnosyl-galactoside: + FBF Spectrum (rt: 31.457-31.805 min) 7.d Subtract
x10 5
757.2133
([C33H40O20]+H)+
Counts vs. Mass-to-Charge (m/z)

Figure (2S). Extracted ion chromatogram and their mass spectrum. (a) A chromatograph of myricetin 3-*O*-arabinoside (Compound 91, Table 2), Retention time (RT = 37.063 min) in the positive mode of ionization ($ESI^+/[M+H]^+$) tentatively identified only in juniper berries; **(b)** Mass spectra of myricetin 3-*O*-arabinoside showing an observed *m*/*z* 451.0850; **(c)** A chromatograph of kaempferol 3-*O*-glucosyl-rhamnosyl-galactoside (Compound 83, Table 2), Retention time (RT = 31.623 min) in the positive mode of ionization ($ESI^+/[M+H]^+$) tentatively identified in both juniper berries and hops extracts; (**d**) Mass spectra of kaempferol 3-*O*-glucosyl-rhamnosyl-galactoside showing an observed *m*/*z* 757.2133.
